# Supplementary material for: Long-lived topological time-crystalline order on a quantum processor
Source: Nat Commun. 2024 Oct 17;15:8963. doi: 10.1038/s41467-024-53077-9 (PMC11487055; doi:10.1038/s41467-024-53077-9)
Supplement: Supplementary file 1 — Supplementary Information [file 41467_2024_53077_MOESM1_ESM.pdf]

Supplementary Information: Long-lived topological time-crystalline order on a quantum processor

Contents

Supplementary Note 1. Theoretical analysis 1
A Surface code model 1
B Topological order of the surface code model 2
C Topological time-crystalline order 3
D Stability of topological time-crystalline order 5
E Perimeter law 8
F Comparison with Floquet symmetry-protected topological phases 8
G Quantum circuits for the Floquet unitary 9
H Floquet eigenstate preparation circuits 10

Supplementary Note 2. Experimental information 11
A Experimental platform 11
B System calibration 12
C Microwave crosstalk 13
D Flux-bias crosstalk 13
E Device-aware circuit transformation 14
F Measurement of auto-correlation functions 17

Supplementary Note 3. Numerical simulations 18
A Error model and noisy simulation 18
B Numerical comparison with echo sequence 19

References 20

Supplementary Note 1. Theoretical analysis

In this work, we experimentally observe topological time-crystalline order, which can be characterized by the subharmonic temporal response of nonlocal logical operators [1, 2]. The topological time-crystalline order is realized in a periodically driven surface code model. In this section, we will first briefly introduce the topological properties of the surface code and provide theoretical analysis of the emergence of topological time-crystalline order. Then, we will show how to use a set of elementary quantum gates to implement the Floquet unitary and to prepare the Floquet eigenstates with a programmable superconducting quantum processor.

A. Surface code model

Recent progress [1, 3, 4] has demonstrated that many physical systems and their properties can be described using the language of topology; an important example of this is topological stabilizer codes [5]. The surface code model is an important topological stabilizer code that has numerous applications in quantum error correction [6–9]. It is analytically solvable and is also of interest to researchers from other fields, including condensed matter physics.

We adapt a variant of the surface code model: the rotated surface code defined on a planar lattice with open boundary conditions [10] (see Supplementary Figure 1). Its Hamiltonian is given by

$$H = -\sum_p \alpha_p A_p - \sum_q \beta_q B_q, \text{ with } A_p = \prod_{k\in p} \sigma_k^z \text{ and } B_q = \prod_{k\in q} \sigma_k^x, \tag{1}$$

where the plaquettes p and q are shown in Supplementary Figure 1, and $\alpha_p,\ \beta_q$ are randomly chosen positive coefficients. For simplicity, unless otherwise specified, we do not distinguish the plaquette operators and the semicircle operators in the following discussions. We note that $A_p,\ B_q$ are both Pauli strings and have eigenvalues ± 1. String operators consisting of the same type of

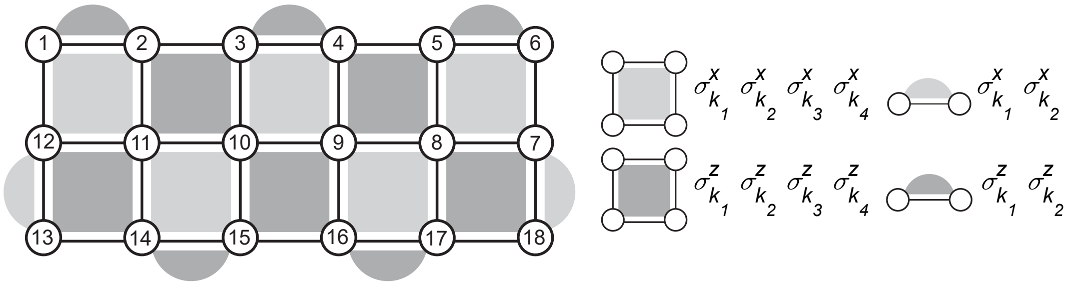

Supplementary Figure 1. **The layout of a 3×6 rotated surface code model.** Circles represent qubits. The shaded plaquettes and semicircles indicate the local four-body and two-body operators on connected qubits, respectively. The dark (light) gray regions represent the A_p (B_q) plaquette operators.

Pauli operators commute with each other: $[A_p, A_{p'}] = [B_q, B_{q'}] = 0$. Additionally, A_p and B_q also mutually commute because the overlap between the support of A_p and any B_q has an even number of qubits. We conclude that the ground states of the rotated surface code model are simultaneous eigenstates of all plaquette operators A_p and B_q with eigenvalue $+1$. Furthermore, each excited eigenstate of the Hamiltonian is a simultaneous eigenstate of all plaquette operators with different eigenvalues. The model in Supplementary Figure 1 has 18 physical qubits and 17 independent plaquette operators, and the remaining single-qubit degree of freedom leads to a two-fold degeneracy for each energy level.

Suppose $|\psi\rangle$ is one of the ground states of H defined on the lattice shown in Supplementary Figure 1. That is, all plaquette operators have an expectation value of 1: $\langle\psi|A_p|\psi\rangle = \langle\psi|B_q|\psi\rangle = +1$ for arbitrary plaquettes p, q. To find another ground state, we try to flip the qubit with index $k = 1$. However, this changes the sign of the expectation value of at least one of the plaquette operators, such as the one supported by qubits 1 and 2, i.e. $\langle\psi|\sigma_1^x A_{\{1,2\}}\sigma_1^x|\psi\rangle = -1$. Thus, the state with one flipped spin is no longer a ground state. To maintain the sign of $\langle A_{\{1,2\}}\rangle$, we flip qubit 2, but this changes the signs of other connected plaquette operators. Thus we are forced to continue this process until qubits $k = 1, \ldots, 6$ are all flipped. The final state is then still a simultaneous $+1$ eigenstate of all plaquette operators, since the string flip operator commutes with all plaquette operators: $\left[\prod_{k=1}^{6}\sigma_k^x, A_p\right] = \left[\prod_{k=1}^{6}\sigma_k^x, B_q\right] = 0$. We denote the string operator by $X_L \equiv \prod_{k=1}^{6}\sigma_k^x$. It cannot be represented as a product of any combination of plaquette operators. Similarly, we can also define $Z_L \equiv \prod_{k=1,12,13}\sigma_k^z$, which anticommutes with X_L. This defines a Pauli algebra on a single logical qubit, and we conclude that the ground state manifold has a two-fold degeneracy indexed by, e.g., $\langle Z_L\rangle = \pm 1$. The same analysis can also be applied to any excited eigenstate. For the l-th energy level, we denote the eigenstates satisfying $\langle Z_L\rangle = \pm 1$ by $|Z_L^{(l)} = \pm 1\rangle$. In the topological stabilizer formalism, plaquette operators are called stabilizers, and the ground space is called the code space, which is manipulated by logical string operators X_L, Z_L.

B. Topological order of the surface code model

In the language of group theory, we can define a group generated by the plaquette operators (stabilizers) $S = \langle A_p, B_q\rangle$, and an 18-qubit Pauli group $P_{18} = \langle \sigma_k^x, \sigma_k^z : k \text{ runs over all sites}\rangle$. S is a subgroup of P_{18}. The centralizer group $C_{P_{18}}(S)$ consists of all operators in P_{18} that simultaneously commute with all plaquette operators. For the rotated surface code model, S is a normal subgroup of $C_{P_{18}}(S)$ and we have the quotient group $C_{P_{18}}(S)/S \cong P_1$ being the Pauli group for one qubit. The above discussion indicates that the two-fold degeneracy of each eigenstate of Supplementary Equation (1), discussed in the previous section, is directly related to the topology of the system. Any single-qubit operator fails to commute with at least one plaquette operator. According to the discussion in Supplementary Note 1.A, we know that Z_L and X_L are two independent nonlocal operators which can map one eigenstate to its degenerate partner. As a consequence, they can be regarded as the representatives of the cosets of S. Since arbitrary products of the plaquette operators with Z_L (X_L) are equivalent to Z_L (X_L) itself in a fixed stabilizer eigenspace, we have several equivalent expressions for operators Z_L and X_L (see Fig. 2 of the main text): $Z_{L_1} = \prod_{k=1,12,13}\sigma_k^z$, $Z_{L_2} = \prod_{k=2,11,14}\sigma_k^z, \ldots$ and $X_{L_1} = \prod_{k=1}^{6}\sigma_k^x$, $X_{L_2} = \prod_{k=7}^{12}\sigma_k^x, \ldots$, all of which are nonlocal operators. All operators within each coset are equivalent modulo S.

A connection between topological order and quantum entanglement is provided by the topological entanglement entropy. For a many-body wavefunction, the von Neumann entropy of a subregion, $S(\rho_{\text{sub}}) \equiv -\operatorname{tr}\rho_{\text{sub}}\ln\rho_{\text{sub}}$, describes the quantum entanglement between the subregion and its complement. Here, ρ_{sub} denotes the reduced density matrix of the subregion obtained by tracing out the complementary region. For a system obeying the entanglement area law, the von Neumann entropy of a

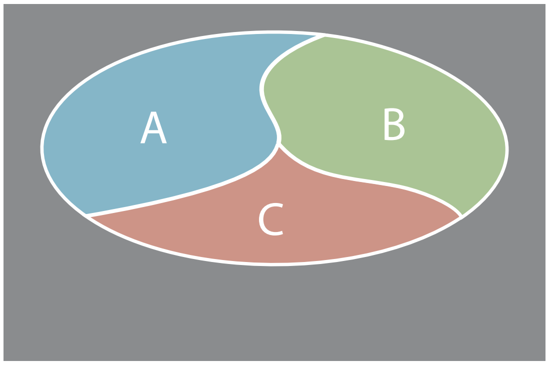

Supplementary Figure 2. Regions A, B, and C defined for computing topological entanglement entropy via Supplementary Equation (3).

subregion is represented as [11]

$$S(\rho_{\text{sub}}) = \alpha \partial_{\text{sub}} - \gamma + \cdots . \tag{2}$$

Here, α is a constant determined by the details of the system, ∂_{sub} is the volume of the subregion's boundary, and $-\gamma$ is the topological entanglement entropy, which characterizes nonlocal entanglement persisting at arbitrarily large distances. In topological quantum field theory, it is known that $\gamma = \ln \mathcal{D}$, where $\mathcal{D}$ is the total quantum dimension [11]. In an Abelian anyon model, $\mathcal{D}$ is the square root of the number of superselection sectors corresponding to inequivalent quasi-particle species. For the rotated surface code model, there are two types of Abelian anyons: electric charges e associated with A_p plaquette operators and magnetic charges m associated with B_q plaquette operators. So, there are four quasi-particle sectors (identity, e, m, and em) and the total quantum dimension $\mathcal{D}$ is equal to $\sqrt{4} = 2$. Thus, the topological entanglement entropy $S_{\text{topo}} = -\ln 2$ for the model [Supplementary Equation (1)]. To measure S_{topo}, we cancel out boundary contributions by dividing the subregion into three parts that are all large compared to the correlation length [11] (see Supplementary Figure 2) and computing

$$S_{\text{topo}} = S_A + S_B + S_C - S_{AB} - S_{BC} - S_{AC} + S_{ABC}, \tag{3}$$

where S_A is the von Neumann entropy of region A, S_{AB} is the von Neumann entropy of region $A \cup B$, and so on. In this way, all boundary terms are canceled out, and the result is the topological entanglement entropy $S_{\text{topo}} = -\gamma$.

C. Topological time-crystalline order

Having reviewed the concept of topological order in the static rotated surface code model, we now generalize to the periodically driven setting and show the emergence of topological time-crystalline order. The Floquet Hamiltonian of the driven rotated surface code model is

$$\begin{aligned} H(t) &= \begin{cases} H_1, & 0 \le t < T', \\ H_2, & T' \le t < T, \end{cases} \\ H_1 &\equiv \frac{\pi}{2} \sum_k \sigma_k^x + \sum_k \boldsymbol{B}_k \cdot \boldsymbol{\sigma}_k, \\ H_2 &\equiv -\sum_p \alpha_p A_p - \sum_q \beta_q B_q, \end{aligned} \tag{4}$$

where $\boldsymbol{B}_k$ is an on-site field randomly chosen from a ball with radius B, $\boldsymbol{\sigma}_k$ is the vector of Pauli operators, A_p, B_q are plaquette operators defined in Supplementary Figure 1, α_p, β_q are coefficients uniformly chosen from $[0, 2\pi)$, and $T = 2T' = 2$. H_2 is the rotated surface code Hamiltonian, and its spectrum is exactly two-fold degenerate, with eigenstates $|Z_L^{(l)} = \pm 1\rangle$ at energy ϵ_l. These two-fold degenerate eigenstates cannot be distinguished by any local operator. However, they can be distinguished by a nonlocal string operator such as Z_L. When $B = 0$, the effect of $U_1 = e^{-i\pi/2 \sum_k \sigma_k^x} \propto \prod_k \sigma_k^x$ is to flip all the qubits. When we index degenerate eigenstates of H_2 with a logical operator Z_L of odd length, this is equivalent to applying a logical operator X_L. In this situation, we have the relations

$$U_F |Z_L^{(l)} = 1\rangle = \exp(-i\epsilon_l) |Z_L^{(l)} = -1\rangle, \quad U_F |Z_L^{(l)} = -1\rangle = \exp(-i\epsilon_l) |Z_L^{(l)} = 1\rangle, \tag{5}$$

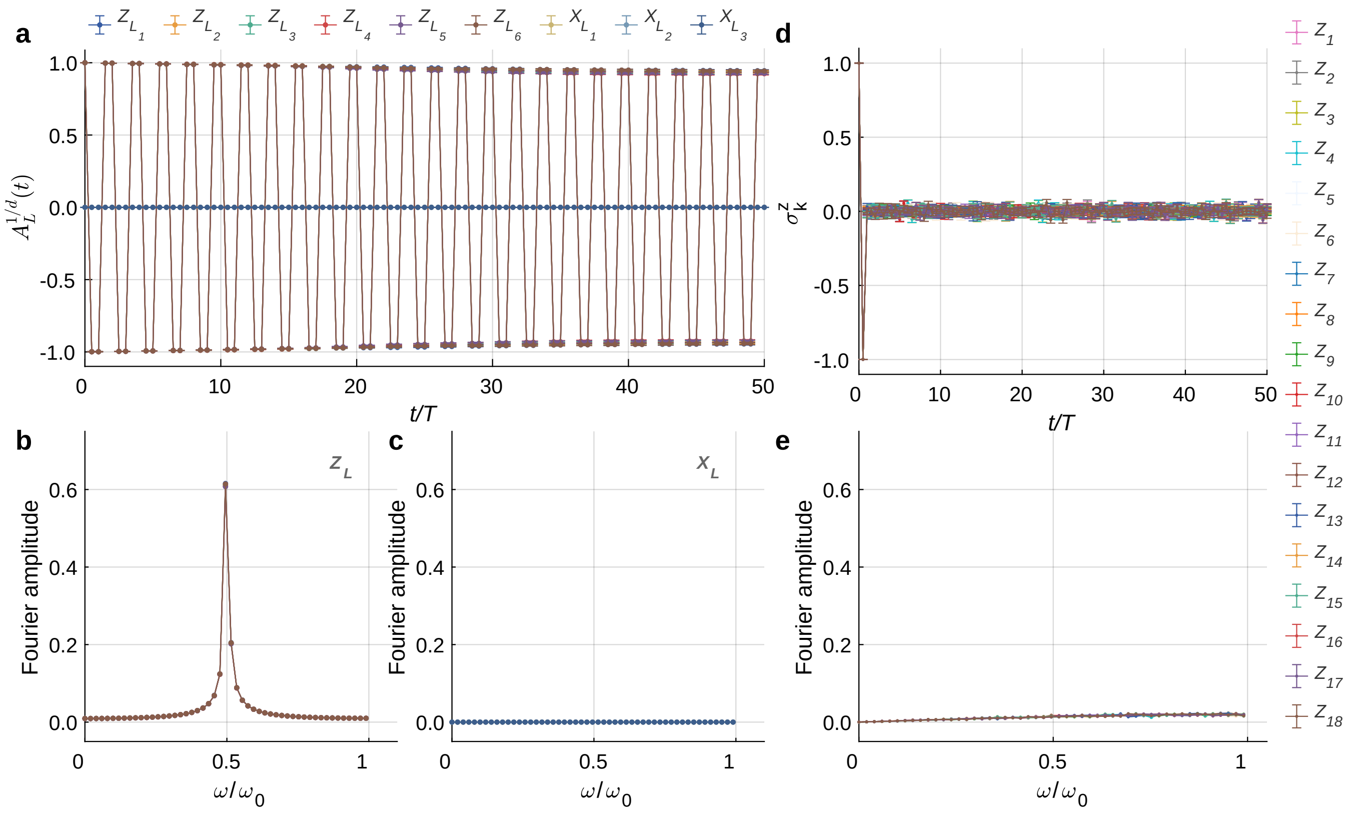

Supplementary Figure 3. **Dynamics of auto-correlation at** $B = 0.1$**.** All data in this figure are averaged over 1000 random realizations, and the error bars stand for the standard error of the statistical mean. **a**, Disorder-averaged dynamics of the auto-correlations for nonlocal string operators $\{Z_{L_i}\}$ and $\{X_{L_i}\}$. Expectation values of all six auto-correlations for $\{Z_{L_i}\}$ lying on top of each other, break the time-translation symmetry, and manifest subharmonic oscillations with period $2T$. Despite the initial slight decay, mainly caused by the imperfect overlap between the dressed logical operators and the measured bare operators, the auto-correlations show a plateau at late times, indicating persistent oscillations. In contrast, expectation values of all three auto-correlations for $\{X_{L_i}\}$ (lying on top of each other) remain zero as expected. **b**, The Fourier spectrum of the disorder-averaged auto-correlation dynamics for $\{Z_{L_i}\}$. The peaks at $\omega/\omega_0 = 1/2$ ($\omega_0 = 2\pi/T$) indicate the subharmonic oscillations of the auto-correlations. **c**, The Fourier spectrum of the disorder-averaged auto-correlation dynamics for $\{X_{L_i}\}$. **d**, Disorder-averaged dynamics of the auto-correlations for single-qubit operators $\{\sigma_k^z\}$. **e**, The Fourier spectrum of the disorder-averaged dynamics of the auto-correlations for single-qubit operators $\{\sigma_k^z\}$.

where $U_F = \exp(-iH_2)\exp(-iH_1)$ is the Floquet unitary. In other words, U_F toggles between two degenerate eigenstates of H_2. In a degenerate eigenspace of H_2, U_F can thus be represented as a matrix

$$U_F \sim \begin{bmatrix} 0 & \exp(-i\epsilon_l) \\ \exp(-i\epsilon_l) & 0 \end{bmatrix}. \tag{6}$$

Therefore, in this eigenspace, U_F has eigenvalues $\pm\exp(-i\epsilon_l)$ corresponding to Floquet eigenstates $|E_\pm^{(l)}\rangle \propto |Z_L^{(l)} = 1\rangle \pm |Z_L^{(l)} = -1\rangle$, respectively. For the Floquet Hamiltonian $H_F = i\log U_F$, the corresponding quasi-energies are ϵ_l and $\epsilon_l + \pi$ (see Fig. 1b of the main text). We note that the H_2 eigenstates $|Z_L^{(l)} = 1\rangle$ and $|Z_L^{(l)} = -1\rangle$ have the same topological entanglement entropy $S_{\text{topo}} = -\ln 2$, which is the core feature of the topological order of this model. The Floquet eigenstates $|E_\pm^{(l)}\rangle$ inherit the same value of S_{topo}.

We further investigate the dynamical behavior of the nonlocal string operators. Without loss of generality, we start from a product state $|\psi_0\rangle$ which has expectation value of $+1$ for the string operator Z_L, such as $|\psi_0\rangle = \bigotimes_k |0\rangle_k$. This can be represented as a superposition of the subset of eigenstates $\{|Z_L^{(l)} = 1\rangle\}$: $|\psi_0\rangle = \sum_l \alpha_l |Z_L^{(l)} = 1\rangle$, such that$\langle\psi_0|Z_L|\psi_0\rangle =$

$\sum_{l'l}\alpha_{l'}^*\alpha_l\langle Z_L^{(l')}=1|Z_L|Z_L^{(l)}=1\rangle=\sum_{l'l}\alpha_{l'}^*\alpha_l\delta_{l'l}=1$. Under time evolution by the Floquet unitary U_F, we find that

$$\begin{aligned} U_F|\psi_0\rangle &= \exp\left(-iH_2\right)\exp\left(-iH_1\right)\sum_l \alpha_l|Z_L^{(l)}=1\rangle \\ &= \exp\left(-iH_2\right)\sum_l \alpha_l|Z_L^{(l)}=-1\rangle \\ &= \sum_l \alpha_l e^{i\epsilon_l}|Z_L^{(l)}=-1\rangle \\ &\equiv |\psi_1\rangle. \end{aligned} \tag{7}$$

If we measure the string operator Z_L after a single Floquet period T, we have

$$\langle\psi_1|Z_L|\psi_1\rangle=\sum_{l'l}\alpha_{l'}^*\alpha_l e^{-i(\epsilon_l-\epsilon_{l'})}\langle Z_L^{(l')}=-1|Z_L|Z_L^{(l)}=-1\rangle=-\sum_{l'l}\alpha_{l'}^*\alpha_l e^{-i(\epsilon_l-\epsilon_{l'})}\delta_{l'l}=-1. \tag{8}$$

Similarly, the stroboscopic dynamics of the string operator Z_L after a time $t=nT$ is $\langle\psi_n|Z_L|\psi_n\rangle=(-1)^n$, where $|\psi_n\rangle=(U_F)^n|\psi_0\rangle$. Therefore, the expectation values of Z_L oscillate with period $2T$, which breaks the discrete time-translation symmetry of the Floquet Hamiltonian [Supplementary Equation (4)]. The corresponding numerical simulations are shown in Supplementary Figure 3a, b. For the string operator X_L, one can check that $\langle\psi_n|X_L|\psi_n\rangle=0$ (see Supplementary Figure 3a, c).

In contrast, we find that local operators exhibit featureless dynamics in our Floquet model. For a single-qubit operator O_{single}, we have

$$\langle\psi_0|O_{\text{single}}|\psi_0\rangle=\sum_{l'l}\alpha_{l'}^*\alpha_l\langle Z_L^{(l')}=1|O_{\text{single}}|Z_L^{(l)}=1\rangle, \tag{9}$$

and

$$\langle\psi_1|O_{\text{single}}|\psi_1\rangle=\sum_{l'l}\alpha_{l'}^*\alpha_l e^{-i(\epsilon_l-\epsilon_{l'})}\langle Z_L^{(l')}=-1|O_{\text{single}}|Z_L^{(l)}=-1\rangle. \tag{10}$$

The extra phase factors $e^{-i(\epsilon_l-\epsilon_{l'})}$ tend to be randomly distributed under the Floquet dynamics of Hamiltonian [Supplementary Equation (4)], leading to a fast decay to zero for $\langle\psi_n|O_{\text{single}}|\psi_n\rangle$ (see Supplementary Figure 3d, e) and thus to an impossibility of time-translation symmetry breaking. This distinction between the dynamics of local and nonlocal operators is the key difference between the topological time-crystalline order and conventional time-crystalline order.

D. Stability of topological time-crystalline order

The stability of the topological time-crystalline order is a highly non-trivial problem and is still open for further exploration. For example, it is believed that many-body localization (MBL) can help prevent a Floquet system from heating to infinite temperature. However, the stability of MBL in the thermodynamic limit is still controversial [12–14] (especially in two or more spatial dimensions), and investigating such open problems is beyond the scope of the present work. Instead, here we provide an argument from the perspective of prethermalization: Time evolving an eigenstate of the Floquet Hamiltonian at $B=0$ with a modified Floquet drive at finite B should not completely destroy the topological order at early times provided B is small. In the limit of high-frequency drive, a Floquet system will enter a long-lived prethermal regime. In this case, the system's dynamics in stroboscopic time can be described by evolution under an effective static Hamiltonian H_{eff}, followed by a perfect spin-flip X,

$$\begin{aligned} U_F &= Xe^{-iH_{\text{eff}}T/2}+O(e^{-1/T}), \\ H_{\text{eff}} &= -\sum_p \alpha_p A_p-\sum_q \beta_q B_q+\sum_k B_{k,x}\sigma_k^x+O(1/T). \end{aligned}$$

Note that X commutes with H_{eff}, allowing the evolution operator between even-numbered periods to be represented as $U(2nT)\approx e^{-iH_{\text{eff}}T}$, which is equivalent to a system evolved under the time-independent H_{eff}. With a small $\boldsymbol{B}$, and hence small $B_{k,x}$, H_{eff} is still topologically ordered, so the evolution of an initially prepared $B=0$ ground state $|E_+^{(0)}\rangle$ within the prethermal regime can be viewed at early times as a weak quench (from $B_{k,x}=0$ to small but finite $B_{k,x}$) within the topologically ordered phase of H_{eff}. At later times but still within the prethermal window, higher-order corrections in B can destabilize the topological order. However, we expect that these corrections become significant on timescales $\gtrsim O(1/B^2)$, such that a finite time window remains in which the state is topologically ordered.

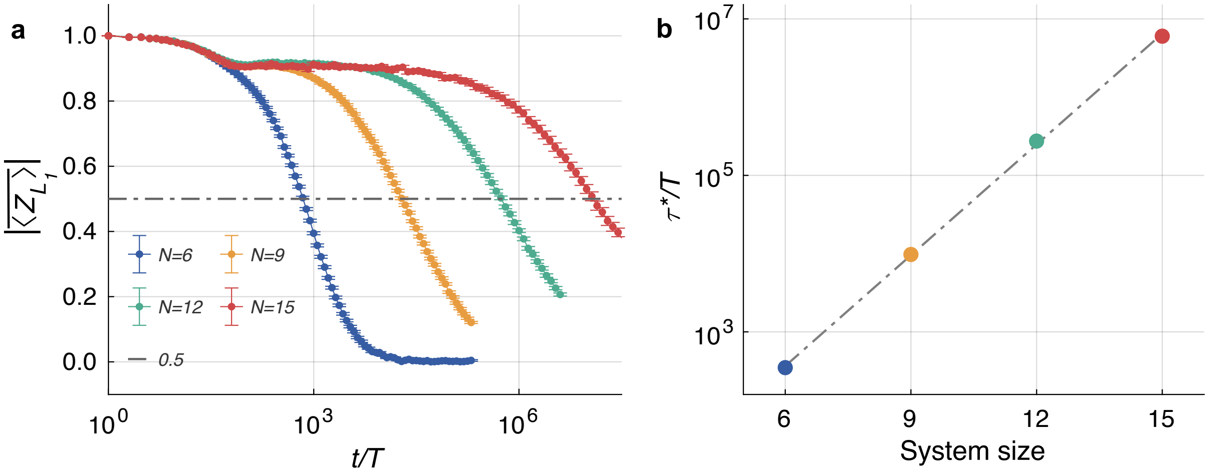

Supplementary Figure 4. **Lifetime of the topological time-crystalline order.** Experimentally measuring the lifetime of the topological time-crystalline order is infeasible due to the limited coherence time of our experimental device. **a**, Disorder-averaged dynamics of the nonlocal logical qubit expectation values $\left|\overline{\langle\psi_n|Z_{L_1}|\psi_n\rangle}\right|$ (we first take the average, and then take the absolute value). The results are averaged over a number of random realizations ranging from 10^3 $(N = 15)$ to 10^4 $(N = 6)$ depending on the system size. The results show that, after an initial slight decay, $\left|\overline{\langle\psi_n|Z_{L_1}|\psi_n\rangle}\right|$ reaches a plateau extending up to a timescale that diverges exponentially with the system size (see panel **b**). The gray dashed line indicates the reference value of $1/2$ that is used to extract the logical-qubit lifetime. Error bars represent the standard error of the statistical mean. **b**, Finite-size scaling of the lifetime of the topological time-crystalline order. The colored dots exhibit the exponential scaling of τ^*/T with system size, where τ^* is the time at which $\left|\overline{\langle\psi_n|Z_{L_1}|\psi_n\rangle}\right|$ reaches $1/2$. The gray dashed line is a best-fit exponential for the system-size dependence of τ^*/T.

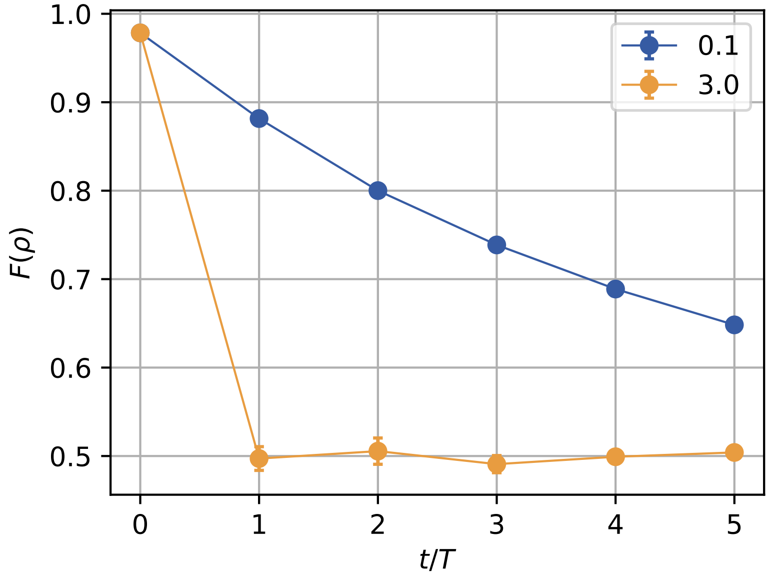

Supplementary Figure 5. Experimentally measured fidelities of the four-qubit subsystem after up to five Floquet drive cycles t/T with small $(B = 0.1)$ and large $(B = 3.0)$ local perturbation strengths. The fidelities are obtained from the same datasets as Fig. 4c of the main text, where $F(\rho)(t \neq 0)$ is obtained by performing state tomography on a four-qubit subsystem and the average is over 12 random realizations; $F(\rho)(t = 0)$ is obtained via the same state tomography process and averaging over five repetitions of eigenstate preparation. Error bars represent the standard error of the statistical mean.

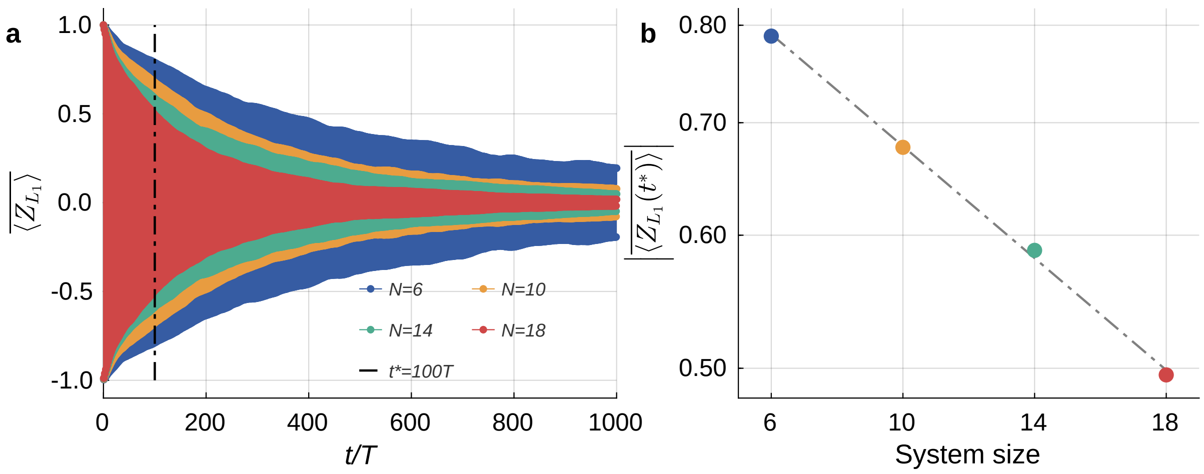

Supplementary Figure 6. **Perimeter law of the time-crystalline order.** **a**, Disorder-averaged dynamics of nonlocal logical operators $\langle\psi_n|Z_{L_1}|\psi_n\rangle$ of different lengths. The results are averaged over 500 random realizations. **b**, Finite-size scaling of the topological time-crystalline order at $t^* = 100T$. Notice that the y-axis is shown on a logarithmic scale. Data points show the exponential scaling of $\left|\overline{\langle Z_{L_1}(t^*)\rangle}\right|$ after a long-time evolution with $t^* = 100T$. Gray dashed line is a best-fit exponential for the system-size dependence of log $\left(\left|\overline{\langle Z_{L_1}(t^*)\rangle}\right|\right)$.

In addition to the above theoretical interpretation, in our experiments, we can also measure state fidelities

$$F(\rho(t)) = \mathrm{tr}\sqrt{\sqrt{\rho(t)}\rho^{\mathrm{ideal}}\sqrt{\rho(t)}}, \tag{11}$$

for the four-qubit subsystem after up to five Floquet cycles (see Supplementary Figure 5). At $B = 0.1$, the disorder-averaged $F(\rho)$ maintains a large value of ~ 0.88 after one period of Floquet evolution and decays slowly with more Floquet cycles, which indicates the topologically ordered initial state $|E_+^{(0)}\rangle$ tends to be maintained. In contrast, when B is large ($B = 3.0$), $F(\rho)$ drops to a floor value of ~ 0.5 immediately after one period of Floquet driving.

These additional analyses, together with the experimental results on topological entanglement entropy in the main text, provide convincing evidence to infer that the observed slow decay of S_{topo}(t) at small B is a reasonable witness of the topological order in the state after Floquet evolution.

We also perform a preliminary numerical simulation to explore the stability of the topological time-crystalline order against small local random fields. In our numerical simulations, we define the lifetime as the number of Floquet periods elapsed before the magnitude of the expectation value of Z_L decays to $1/2$. We fix a small perturbation strength $B = 0.1$ for this study. In the presence of random on-site fields, the contribution of H_1 to the Floquet unitary is no longer a perfect spin-flip operator for all the sites; rather, it is

$$U_1 = e^{-iH_1} \sim \prod_k \sigma_k^x + O(B). \tag{12}$$

As the underlying lattice structure can have a significant effect on the lifetime of the logical qubit, we carry out the numerical simulations for lattices of dimensions $3 \times 2, 3 \times 3, 3 \times 4, 3 \times 5$, and measure the non-local logical operator Z_L of fixed length 3. The initial states are randomly chosen z-basis product states in order to give definite expectation values of ± 1 for the string operator Z_L. For each realization, we numerically calculate the dynamics under Supplementary Equation (4) for a long enough time to observe the decay of the string operator. The final results are averaged over many such random realizations, and the time at which the expectation values reach $1/2$ is regarded as the indicator of the lifetime (see Supplementary Figure 4a). Our results show that the lifetime of the topological order for the model is much longer than any experimentally accessible timescale. Furthermore, we observe that the lifetime of the logical qubit grows exponentially with system size for $N = 6, 9, 12, 15$ (see Supplementary Figure 4b). We conclude that, owing to disorder, the true thermalization time is much larger than these experimentally inaccessible timescales, and thus the observed topological time crystalline behavior is robust.

E. Perimeter law

The subharmonic response of a nonlocal logical operator decays [2] as

$$\sim 2^{-\xi l}, \tag{13}$$

where ξ is the localization length and l is the length of the nonlocal logical operator. Intuitively, this can be interpreted from the perspectives of non-contractility and localization. If the system is many-body localized, the original logical operator will still interact with those quasi-particles within the localization length ξ. The number of involved quasi-particles scales with the area ξl covered by the fattened operator. Such interactions cause the spread of the initial information contained in the support of the nonlocal operator, and thus the exponential decay of the time crystalline order. We remark that this exponential decay as a function of l is an incarnation of the perimeter law for Wilson loop operators in lattice gauge theory [15, 16].

A feasible experimental measurement of the perimeter-law scaling requires longer coherence time as well as higher gate fidelity, beyond the reach of currently available NISQ devices. To this end, we perform numerical simulations. The late-time dynamics of nonlocal operators for different system sizes are shown in Supplementary Figure 6. We fix a boundary with a length of 2, and vary the other boundary, labeled by l. Therefore, the system size is $N = 2l$ and the length of logical operator Z_L along the long boundary is l, where $l = 3, 5, 7, 9$. Indeed, we find that after a long-time evolution,

$$\log\left(|Z_L(l)|\right) \propto l$$

This exponential correspondence agrees well with the theoretical analysis above.

F. Comparison with Floquet symmetry-protected topological phases

Our work shows a time crystalline dynamics in the periodically driven surface code, where intrinsic topological order is a critical feature. This novel phase evidently distinguishes itself from many previous works in various aspects. Here, we summarize several important differences between the phase of matter studied in this work and the Floquet symmetry-protected topological phases studied in, e.g., Ref. [17].

First, fundamentally, symmetry-protected topological states and topologically ordered states belong to different quantum phases, and exhibit fundamentally distinct properties:

- Stability: symmetry-protected topological phases require the presence of a symmetry to be defined; as a result, any symmetry breaking terms immediately destroys the symmetry-protected topological phase. By contrast, topologically ordered phases are robust to any local perturbations and do not require any underlying symmetry to be well-defined. A distinct perspective on this point comes in terms of the preparation of such states: while a topologically ordered state can only be generated with a unitary circuit whose depth scales with the system size; for the symmetry-protected topological state, this is only true if all the gates respect the underlying symmetry, and thus shorter preparation schemes exist if the symmetry is not respected during the preparation. For this reason, topologically ordered state is refereed to having long-range entanglement, whereas symmetry-protected topological state has only short-range entanglement.

- Nature of ground state degeneracy: While both phases exhibit a ground state manifold whose degeneracy depends on the topology of the system itself, their origin is very different. In the case of an symmetry-protected topological phase, the symmetry acts in a non-trivial way in the system such that it ensures the existence of localized gapless edge modes. For example, in the one-dimensional spin-1 AKLT system, this manifests itself as the existence of two decoupled spin-1/2 degrees of freedom in the edge. As such, different ground states can be accessed by acting with local operators on the edge. By contrast, in a topologically ordered phase, the degeneracy of the ground state is not just encoded in the edge degrees of freedom. One can see that in terms of the operators required to transform between the different ground states: the simplest example (which is of relevance to our work as well) is the toric code where the necessary string operators span the entire system size.

Second, for Floquet symmetry-protected topological phases, only local edge spins show subharmonic response. However, for topologically ordered time crystals, only non-local string operators show period-doubling dynamics. While string order parameters can also diagnose the presence of symmetry-protected topological phases, its oscillation period is the same as the driving period, and no subharmonic response will be observed. The period-doubled dynamics of nonlocal string operations is also one of the key differences between the topologically ordered time crystal and previously studied conventional time crystals, as we stated in the main text.

Third, the "perimeter law" of the non-local order parameter is a unique feature of the topologically ordered time crystal, and it has no counterpart for Floquet symmetry-protected topological phases. As stated in the previous section, the late-time

	Phases	Measurability	Perimeter law	Stability	Entanglement strcuture
Topologically ordered time crystal	TO	Non-local operator	Yes	Arbitray perturbation	Long-range entanglement
Floquet SPT phases	SPT	Local operator	No	Symmetric perturbation	Short-range entanglement

Supplementary Table 1. Comparison between the topologically ordered time crystal and Floquet symmetry-protected topological phases.

value of the non-local logical operator Z_L will exponentially decay to the length of the corresponding string [2]. Bynumerically simulating the dynamics of the topologically ordered time crystal with different system sizes, we showed in Supplementary Figure 6 that the late-time envelopes of the non-local operators Z_L agree well with the perimeter law. This also distinguishes the topologically ordered time crystal from previously studied Floquet symmetry-protected topological phases [17] in term of measurability.

Fourth, the two dynamical phases are distinct in term of stability. Floquet symmetry-protected topological phases are only stable to local perturbations respecting the protecting symmetries. Asymmetric perturbations will mix eigenstates at different quasienergies and thus wash out the non-trivial topological features. However, the eigenstates of topologically ordered time crystal are long-range entangled and the degeneracy in the quasienergy spectrum is fully determined by the topology of the manifold on which the system is defined. This leads to absolute stability against arbitrary local perturbations.

Fifth, the Floquet eigenstates for Floquet symmetry-protected topological phases and the topologically ordered time crystal have different entanglement structure. The topological entanglement entropy quantifies the long-range entanglement of a state [11, 18]. Floquet eigenstates for Floquet symmetry-protected topological phases are short-range entangled and have zero topological entanglement entropy. However, Floquet eigenstates for topologically ordered time crystals are long-range entangled as demonstrated experimentally by our measurement of a non-zero topological entanglement entropy (see Fig. 3 in the main text).

We summarize these differences in Supplementary Table 1.

G. Quantum circuits for the Floquet unitary

It is straightforward to realize the Floquet drive $U_1(t) = e^{-itH_1}$ with tensor products of single-qubit rotations, which can be represented via Euler angles (see Supplementary Figure 7a). However, the circuit construction of $U_2(t) = e^{-itH_2}$ is more challenging due to the four-body plaquette operators A_p, B_q in the Hamiltonian H_2. To construct digital quantum circuits for this evolution, we exploit the property that all plaquette operators mutually commute, so that the evolution reads

$$U_2(t) = e^{-itH_2} = \prod_p e^{it\alpha_p A_p} \prod_q e^{it\beta_q B_q}. \tag{14}$$

Besides, we have the relation $H\sigma^z H = \sigma^x$, where H is the single-qubit Hadamard gate. Thus, for any plaquette q, we have

$$H^{\otimes q} \left(e^{it\beta_q A_q}\right) H^{\otimes q} = e^{it\beta_q B_q}, \tag{15}$$

where $H^{\otimes q}$ stands for a Hadamard transform applied to the qubits in plaquette q. Therefore, the circuit construction for evolution under H_2 reduces to simulating a single plaquette operator A_p.

Variational quantum circuits are a powerful tool for NISQ quantum computation and quantum simulation and have been intensively studied in recent years [19, 20]. We adapt this method to construct the quantum circuit for evolution under the plaquette operator A_p. Variational quantum circuits are composed of gates with parameterized rotation angles that can be updated according to various algorithms. The circuit construction for the evolution operator of A_p can be divided into two steps. First, we need to find an appropriate variational ansatz for it. Second, we optimize the variational parameters in this ansatz to minimize the distance between the corresponding quantum circuit and the target unitary.

For the first step, we use the neuroevolution method [21] to find a suitable variational quantum circuit architecture. The complete gate set used in our experiment consists of three kinds of single-qubit rotations $X(\theta), Y(\theta), Z(\theta)$ and a controlled-phase gate $\mathrm{CR}_z(\theta)$ along the z axis (θ stands for the variational parameter). Then, we can construct a directed graph where each node represents a block of gates that can be implemented in parallel, and where the directed edges denote allowed sequences of blocks. A quantum circuit can then be represented as a directed path in this graph. To find a desired circuit ansatz, we use the following procedure:

1. Randomly sample several paths with a fixed depth in the constructed directed graph as our initial quantum-circuit ansatz;

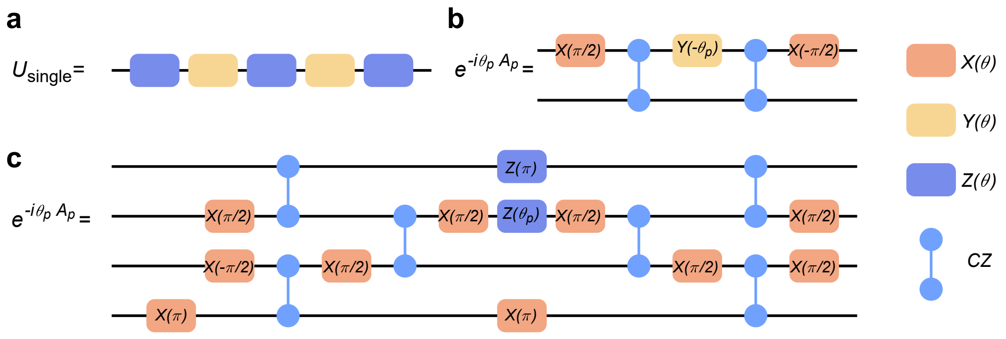

Supplementary Figure 7. **Digital quantum circuits for the Floquet unitary.** (**a**) Single-qubit circuit realizing U_1. We use Euler angles to represent general single-qubit rotations. (**b**) Circuit realizing the evolution unitary of the two-body plaquette A_p on the boundary of the lattice (see Supplementary Figure 1). (**c**) Circuit realizing the evolution unitary of the four-body plaquette A_p. Combining these elementary circuits allows us to digitally simulate the Hamiltonian [Supplementary Equation (4)]. In experiments, the whole circuit is further compiled to reduce the depth.

2. For each path representing an ansatz, minimize the cost function $L(\boldsymbol{\theta})$, which is given by

$$L(\boldsymbol{\theta}) = 1 - \mathrm{Tr}\left[U^{\dagger}_{\text{target}}\, U_{\text{circuit}}\left(\boldsymbol{\theta}\right)\right]/d, \tag{16}$$

where U_{target} is the evolution unitary of the plaquette operator A_p, U_{circuit} is the unitary represented by the current quantum-circuit ansatz with variational parameters $\boldsymbol{\theta}$, and d is the dimension of the Hilbert space. $L(\boldsymbol{\theta})$ measures the distance between the target unitary and the current quantum circuit. We update variational parameters using gradient-based algorithms;

3. Keep the ansatzs with smallest values of the loss function $L(\boldsymbol{\theta})$, and prolong the corresponding paths in the directed graph to generate new circuits with larger depth;

4. Iterate steps 2 and 3 until the loss function converges.

We repeat this algorithm several times and choose the circuit ansatz with the smallest value of the loss function as the optimal result.

Remarkably, in some cases, we can obtain ansatz circuits with an extremely small distance from our target unitary (typically, the loss function $L(\boldsymbol{\theta}) < 10^{-4}$), which indicates that there probably exists a circuit ansatz that is analytically equivalent to the target unitary. Thus, we further manually simplify the obtained variational circuit ansatz. Precisely speaking, we alternately utilize the following methods to reduce the number of the variational parameters in this ansatz:

1. Drop those gates very close to the identity gate, i.e., variational gates with small rotation angles.

2. Fix those gates with special parameters, such as $\theta = \pi$.

3. Change the order of some commuting gates.

4. Split or combine some neighboring gates.

After the reduction process above, we obtain an experimentally friendly circuit that analytically represents the evolution unitary $U(t) = e^{it\alpha_p A_p}$ (see Supplementary Figure 7b, c). The evolution unitary of B_q is then obtained by inserting a layer of Hadamard gates before and after $U(t)$. Then, we can obtain the digital quantum circuit for U_2 by concatenating the evolution operators for all plaquette operators. This yields an analytical representation of the whole time-evolution unitary (see Fig. 1 of the main text).

H. Floquet eigenstate preparation circuits

In the main text, we experimentally measure the topological entanglement entropy of a Floquet eigenstate. Here, we provide more details on the circuit for preparing this state. In our model, the Floquet eigenstates $|E^{(l)}_{\pm}\rangle \propto |Z^{(l)}_L = 1\rangle \pm |Z^{(l)}_L = -1\rangle$ are superpositions of the eigenstates ($|Z^{(l)}_L = \pm 1\rangle$) of the rotated surface code Hamiltonian. We follow the method in Ref. [22] to realize the eigenstates $|Z^{(l)}_L = \pm 1\rangle$ and their superpositions. Here, we briefly summarize the idea of this method.

Without loss of generality, we choose the Floquet eigenstate that superposes the two-fold degenerate ground states of H_2. Because the ground states are the simultaneous eigenstates of all plaquette operators, we can apply the projector $\prod_{p,q}(\mathbb{1}+A_p)(\mathbb{1}+B_q)$ to map the initial state into the ground space. For simplicity, we choose an initial state of $|0\rangle^{\otimes 18}$. The projector above is a sequence of mutually commuting projection operators, which project the initial state into a simultaneous eigenstate of A_p and B_q. The final state is unchanged after exchanging the order of the projection operators, since $[A_p, B_q] = 0$. Since $(\mathbb{1}+A_p)|0\rangle^{\otimes 18} = |0\rangle^{\otimes 18}$, we conclude that $|Z_L^{(0)} = 1\rangle \propto \prod_q(\mathbb{1}+B_q)|0\rangle^{\otimes 18}$ (ignoring an overall normalization factor). Furthermore, since $|Z_L^{(0)} = -1\rangle = X_L|Z_L^{(0)} = 1\rangle$, we can express the superposition of ground states in two topological sectors as $|Z_L^{(0)} = 1\rangle + |Z_L^{(0)} = -1\rangle \propto (\mathbb{1}+X_L)\prod_q(\mathbb{1}+B_q)|0\rangle^{\otimes 18}$. Since B_q only consists of σ^x operators, we have $[X_L, B_q] = 0$. As a result, we can also write $|Z_L^{(0)} = 1\rangle + |Z_L^{(0)} = -1\rangle \propto \prod_q(\mathbb{1}+B_q)(\mathbb{1}+X_L)|0\rangle^{\otimes 18}$.

Since all the X_{L_k} are equivalent in our model, we fix $X_L = \prod_{k=7}^{12}\sigma_k^x$ in the following discussion. We first show how to prepare the state $(\mathbb{1}+X_L)|0\rangle^{\otimes 18}$. The effect of $\mathbb{1}+X_L$ is to project the initial zero state into a cat state of the form$(|000000\rangle + |111111\rangle)_{7,\ldots,12} \otimes |0\rangle^{\otimes 12}$. This cat state can be prepared conveniently with quantum gates by first applying a Hadamard gate on one of the qubits in $\{7,\ldots 12\}$ and then successively applying CNOT gates for each pair of neighboring qubits. The effect of the projection operators $\mathbb{1}+B_q$ can be realized by applying similar gate sequences to the qubits in plaquette q.

Supplementary Note 2. Experimental information

Exploring an intrinsically non-equilibrium Floquet system [Supplementary Equation (4)] relies on the dynamical manipulation of highly entangled many-body states. In our work, we engineer a topological "synthetic quantum material" on a 3×6 superconducting qubit lattice using the digital quantum simulation paradigm. In this section, we provide detailed information on the experimental platform, device performance, and circuit calibration.

A. Experimental platform

As shown in Supplementary Figure 8a and b, we use a 3×6 qubit lattice on a flip-chip superconducting quantum processor to implement the theoretical model. This processor has a two-dimensional architecture consisting of a 6×6 qubit array where the nearest-neighbor (NN) qubit pairs are connected by tunable couplers [23]. Thus, the interaction between each NN qubit pair can be tuned dynamically by applying control signals to the coupler (Supplementary Figure 8c), which enables the implementation of two-qubit CZ gates in our experiments. Each qubit is capacitively coupled to a readout resonator for dispersive readout, and each group of nine readout resonators shares a common readout transmission line for simultaneous state measurements. Supplementary Figure 8c shows the details of the experimental setup, including wiring information, microwave components, and room-temperature control electronics. The performance of our quantum computing hardware is significantly improved

Supplementary Table 2. Hardware improvements exploited in this study compared with the previous work [17].

Hardware parameters	Ref. [17]	This work
T_1 (median)	33.0 μs	162.6 μs
Maximum circuit length	10.4 μs	28.8 μs
Two-qubit gate connectivity	1D chain	2D lattice
Single-qubit gate error	0.55×10^{-2}	0.48×10^{-3}
CZ gate error	1.57×10^{-2}	0.64×10^{-2}
Number of CZ layers	160	420
Number of SQ layers	80	300
DAC board waveform storage	$\sim$ 15 μs	$\sim$ 130 μs
DAC resolution	14 bits	16 bits
DAC sampling rate	1×10^9 Hz	2×10^9 Hz

relative to our previous work [17], which is crucial to enable this challenging experiment. Simulating the topologically ordered time crystal requires ~700 layers of quantum gates (see Supplementary Note 2.E) on a 2D array, which is about 3 times more

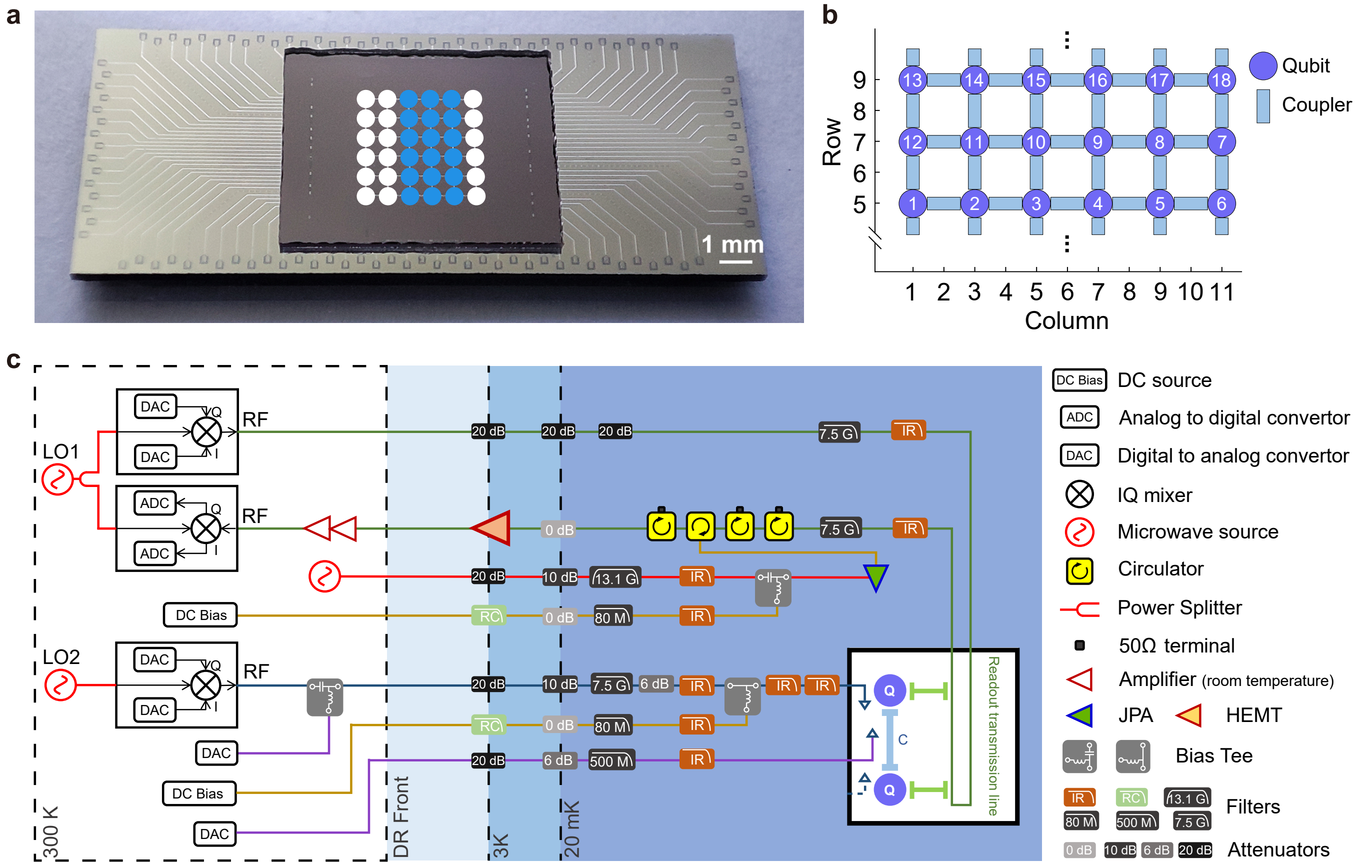

Supplementary Figure 8. **Quantum processor and experimental setup.** **a**, Photo of the flip-chip quantum processor. The 18 qubits actively used in our experiments are marked by blue solid circles, while the other unused qubits are marked by white circles. **b**, Schematic structure of the 3×6 qubit lattice. Qubits are arranged at the vertices of a $3{\times}6$ grid, and couplers are represented by the edges. **c**, Wiring information. The superconducting quantum chip is mounted on the mixing chamber plate (20 mK) of the dilution refrigerator. For simplicity, we use a square box at the bottom right corner, wherein a pair of qubits is coupled to a coupler, to represent the chip. To control and measure the chip, qubits and couplers are connected to the room-temperature electronics by readout lines (green), microwave-drive lines (blue), fast Z-pulse lines (purple) and slow DC-bias lines (brown). Information on the microwave components is provided in the legend on the right.

than the previous work ($\sim$240 layers in a 1D chain). Therefore, it is necessary to improve coherence time, control electronics, and gate fidelity so that we can observe the topologically ordered time crystal before the accumulated circuit errors dominate the system dynamics and totally destroy the coherent quantum behavior. We summarize the improvements of quantum computing hardware in Supplementary Table 2. Compared to our previous work, in this work, the energy relaxation time T_1 is $\sim$5 times longer, two-qubit gate errors are $\sim$3 times lower, single-qubit gate errors are $\sim$10 times lower, the sequence length is $\sim$ 3 times longer, and the DAC capabilities are also largely improved. Putting all these improvements together has been a challenging experimental achievement that enables the study of the topologically ordered time crystal.

B. System calibration

Realizing a well-specified Hamiltonian with digital quantum circuits is a challenging experimental task. It is a complex control problem, whose target is to find optimal parameters which convert time-dependent room-temperature microwave signals to an effective Hamiltonian on the quantum chip at low temperature. In our experiment, we coherently control 45 quantum elements (18 qubits and 27 couplers) for manipulating the topologically ordered Floquet system. Here, we briefly describe our calibration procedures for tuning up these elements.

Before calibrating the universal quantum gate set (single-qubit rotations and two-qubit CZ gate), we use the following procedures to get an overall characterization of the device.

1. Perform spectroscopy measurements for each qubit to obtain the relationship between the qubit frequency ω_q and the amplitude of its fast Z pulse.

2. Tune up each qubit individually at a flux-sensitive point, which we choose ∼300 MHz below its maximum frequency (sweet point) in our experiments, and perform a series of measurements to obtain the following basic parameters.
   - Single-qubit π and $\pi/2$ pulse parameters.
   - The ratio between the fast Z-pulse amplitude and the slow DC-bias amplitude. With this ratio, we can further get the relationship between qubit frequency ω_q and the amplitude of the DC bias.
   - Qubit readout pulse parameters.
   - Spectrum of T_1. We note that the T_1 spectrum is repeatedly monitored on different days to detect possible moving two-level-system (TLS) defects [24].
3. Synchronize the timing of the control pulses from different control lines. We select the center qubit as the root and use the Dijkstra algorithm to traverse all the qubits and couplers from near to far. For the detailed calibration pulse sequences, see Ref. [25].
4. Calibrate the distortion of the fast Z pulse. Distortion information is obtained by probing the time-domain response of the qubit phase right after a Z pulse [26]. We note that the distortion of the fast Z pulse for the coupler is derived with the help of the phase response of its adjacent qubits.

With the information above, we can start to tune up the universal quantum gate set on the 3×6 lattice, which includes 18 single-qubit rotations and 27 two-qubit CZ gates. This is challenging due to the existence of pulse distortions, TLS defects, and crosstalk. We allocate a set of idle frequencies $\{\omega_{10}\}$ to qubits, which are optimized to yield high-fidelity single-qubit gates as well as to favor the implementation of two-qubit CZ gates. We consider several important principles, which are listed below.

- Energy relaxation time T_1 and spin-echo pure dephasing time T_2^{SE} in the vicinity of ω_{10} should be long and stable.
- Frequency detuning between two qubits with stray coupling should be much larger than the strength of the stray coupling.
- The fast Z-pulse amplitude for realizing a two-qubit CZ gate should be small to minimize the impact of residue pulse distortion.

In each optimization round, we tune up all the single- and two-qubit gates, and then test their fidelities by performing simultaneous cross-entropy benchmarking (XEB) [27] using the typical layers in the target circuits. These results are used as the feedback for the next round of optimization. After several rounds, we obtain a set of idle frequencies and gate parameters for our experiments. The idle frequencies $\{\omega_{10}\}$ in this experiment are shown in Supplementary Figure 9a. The measured energy relaxation time T_1 and spin-echo dephasing time T_2^{SE} at $\{\omega_{10}\}$ are listed in Supplementary Figure 9b and c, respectively. Their cumulative distributions and median values are shown in Supplementary Figure 9d, e, and f. Remarkably, the median value of T_1 over 18 qubits is ∼163 μs. We also achieve median Pauli errors (ϵ_p) of $\sim 0.48 \times 10^{-3}$ for single-qubit gates and $\sim 6.4 \times 10^{-3}$ for two-qubit CZ gates, which is equivalent to the randomized-benchmarking fidelities [28] of 0.9997 and 0.9949, respectively. Here we use the relation $F = 1 - \epsilon_p/(1 + 1/2^d)$, where d is the number of qubits. Supplementary Figure 10 shows detailed information on gate errors.

Readout fidelities of qubits are simultaneously measured by preparing 18 qubits in random product states [29] and averaging them for each qubit. Supplementary Figure 11 displays the measured readout fidelities for each qubit in our experiment, which are also used to correct the effects of readout errors.

C. Microwave crosstalk

Single-qubit rotations for each qubit are realized by applying microwave pulses to its microwave drive line. However, microwave pulses applied to Q_i will also induce unwanted state transitions of Q_j. The microwave pulse crosstalk felt by Q_j can be modeled as $\tilde{\Omega}_j = A_{ji} e^{-i\phi_{ji}} \Omega_i$ [30], where Ω_i is the microwave signal applied to the source qubit Q_i, while $A_{ji} e^{-i\phi_{ji}}$ describes the relative amplitude and the relative phase of the effect on the target qubit Q_j. It can be canceled by actively applying an opposite signal $-\tilde{\Omega}_j$ to the qubit Q_j. We use randomized benchmarking to detect microwave crosstalk, and use the measured matrix $\{A_{ji} e^{-i\phi_{ji}}\}$ to suppress such effects.

D. Flux-bias crosstalk

In our device, applying a bias current to the flux line of qubit Q_i (or coupler C_i) can cause a nonzero flux on other qubits (or couplers). During parallel gate operations, this flux crosstalk can introduce extra phase errors into the circuit. The crosstalk can

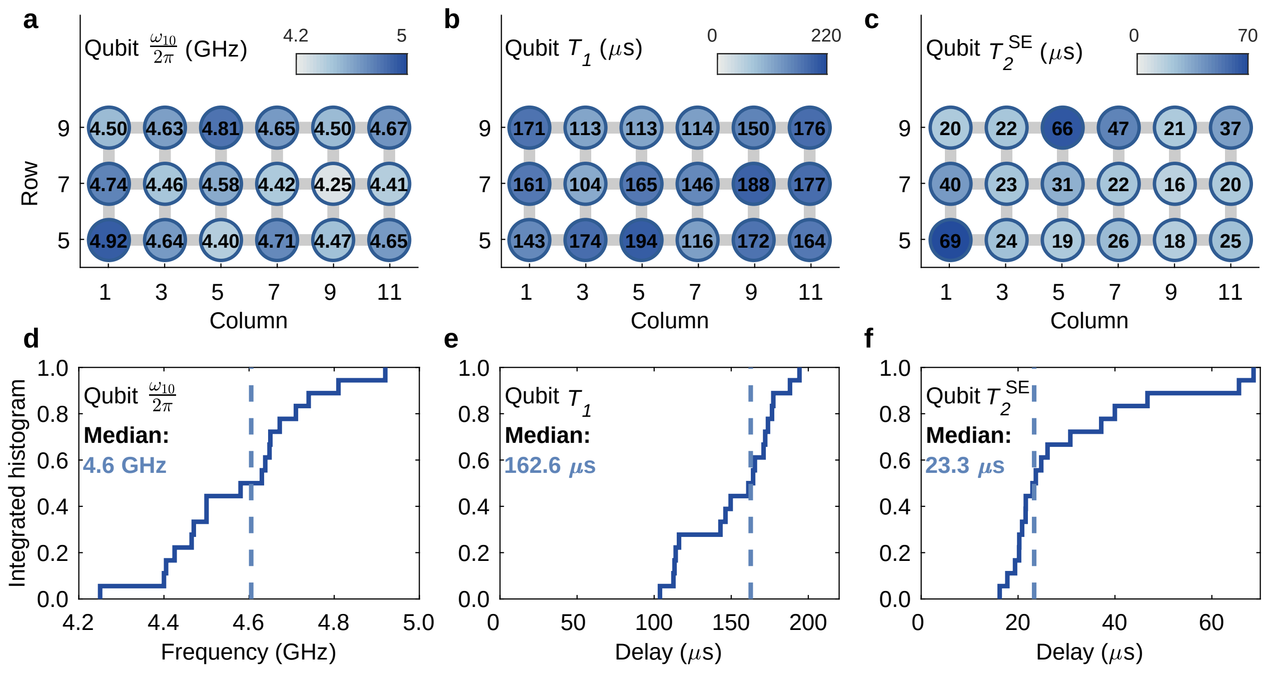

Supplementary Figure 9. **Qubit performance at idle frequencies.** **a**, Heat map of idle frequencies $\{\omega_{10}\}$. **b**, Heat map of qubit energy relaxation times $\{T_1\}$ at idle frequencies. **c**, Heat map of qubit spin-echo pure dephasing times $\{T_2^{\mathrm{SE}}\}$ at idle frequencies. **d**, **e**, and **f** are the integrated histograms of $\{\omega_{10}\}$, $\{T_1\}$ and $\{T_2^{\mathrm{SE}}\}$, respectively, which are obtained using the data in **a**, **b**, and **c**.

be modeled as $\tilde{\Delta}_j = \Delta_i B_{ji}$, where Δ_i is the flux to qubit Q_i (or coupler C_i), $\tilde{\Delta}_j$ is the crosstalk flux felt by qubit Q_j, and B_{ji} is the crosstalk ratio. We neglect crosstalk to couplers in our experiments. To compensate for $\tilde{\Delta}_j$, we measure the ratio B_{ji} and apply a flux bias $-\tilde{\Delta}_j$ to Q_j. The measured crosstalk matrix elements $\{B_{ji}\}$ for fast Z bias are shown in Supplementary Figure 12.

E. Device-aware circuit transformation

A quantum circuit constructed theoretically usually incorporates little information about the limitations or imperfections of the underlying hardware performance, leaving space for further improvements before it is converted to physical control pulses. Therefore, it is worthwhile to transform the circuits generated in Supplementary Note 1 with the awareness of the device information to further improve the circuit fidelity. Supplementary Figure 13 shows the transformed circuit of the Floquet-evolution unitary for a single period T at $B = 0.1$. We summarize the strategies and tools we use in this process below.

1. Remove redundant Clifford gates using ZX-calculus [31, 32].

2. Use Qiskit [33] to convert the circuit into combinations of two-qubit CZ gates and single-qubit rotations around the x, y, z-axis on the Bloch sphere and Cirq [34] to identify single-qubit layers and separate them from CZ gates. Then we get a circuit that alternates between layers of single-qubit and CZ gates: a single-qubit gate layer (SQ layer), followed by a layer of CZ gates (CZ layer), followed by an SQ layer, etc.

3. Compile consecutive SQ gates into a U3 gate. The U3 gate is constructed by combining a θ-angle rotation around the z-axis followed by an α-angle rotation around an axis in the xy plane with an azimuthal angle ϕ, which can be written in the form

$$\mathrm{U3}(\alpha,\phi,\theta) = R_{xy}(\alpha,\phi)R_z(\theta) = \begin{bmatrix} \cos\left(\frac{\alpha}{2}\right) & -ie^{-i\phi}e^{i\theta}\sin\left(\frac{\alpha}{2}\right) \\ -ie^{i\phi}\sin\left(\frac{\alpha}{2}\right) & e^{i\theta}\cos\left(\frac{\alpha}{2}\right) \end{bmatrix}. \tag{17}$$

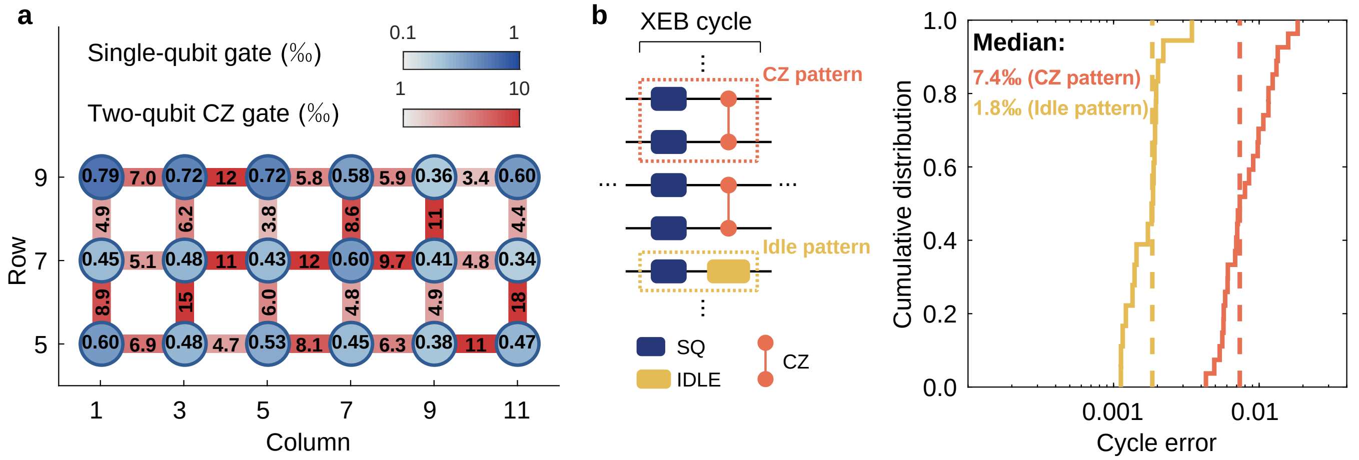

Supplementary Figure 10. **Gate errors.** **a**, Heat map of layer-averaged Pauli errors for single-qubit gates (blue) and two-qubit CZ gates (red). These values are measured by performing simultaneous XEB using the typical single-qubit gate layers (SQ layers) and two-qubit CZ gate layers (CZ layers) in the Floquet unitary circuit at $B = 0$ and in the eigenstate preparation circuit. Note that, for the simultaneous XEB of each CZ layer, those qubits that are not involved in CZ gates undergo a single-qubit XEB sequence. **b**, Schematic of the XEB circuit for CZ layers and cumulative distributions of the cycle error. The left panel shows the XEB circuit for the CZ layer. Each cycle is composed of a single-qubit gate layer and a subsequent CZ layer, which includes two types of patterns, a CZ pattern and an idle pattern. The right panel shows the cumulative distributions of cycle errors for the CZ pattern (red) and the idle pattern (yellow). Each data point represents a specific pattern for the target gate, which is averaged over all CZ layers. Using cycle errors, we can estimate CZ gate errors in **a** and idle gate errors in the CZ layers.

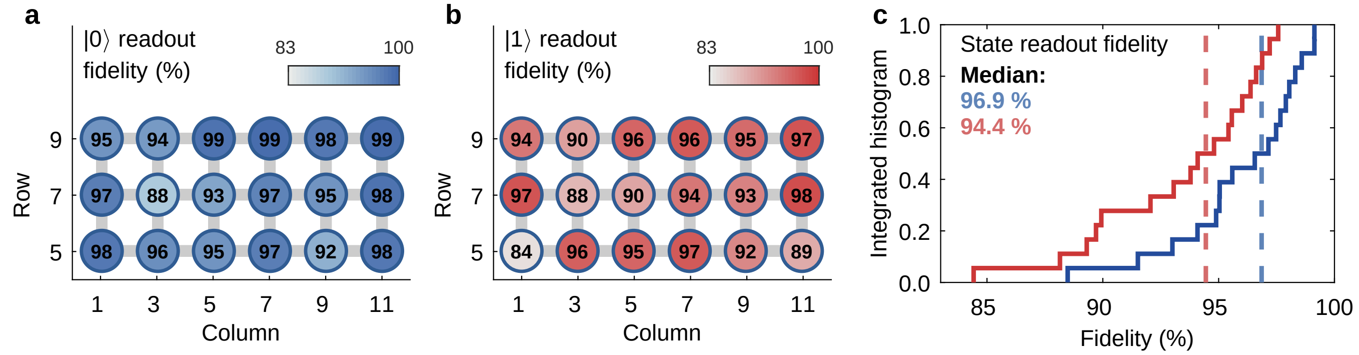

Supplementary Figure 11. **Qubit readout fidelity.** **a**, Heat map of the readout fidelity for state $|0\rangle$. **b**, Heat map of the readout fidelity for state $|1\rangle$. **c**, Integrated histogram of 18 readout fidelities based on the data in **a** and **b**. To obtain the readout fidelities, we prepare all the qubits in random computational-basis product states and perform simultaneous measurements. For each qubit, its $|0\rangle$ ($|1\rangle$) readout fidelity is given by calculating the $|0\rangle$ ($|1\rangle$) state probability from the samples that this qubit is prepared in $|0\rangle$ ($|1\rangle$).

Note that $R_z(\theta)$ is implemented virtually by adding an extra θ to the phase of the subsequent microwave pulse [35]. $R_{xy}(\alpha, \phi)$ is implemented by applying a microwave pulse, whose phase is ϕ and whose amplitude depends on the rotation angle α.

4. Separate CZ gates in a given layer into several groups. This step is to avoid leakage caused by qubit level crossings while multiple CZ gates operate in parallel. A maximum of two groups are enough for our Floquet-evolution circuit.

5. In the eigenstate circuit, we align the gates to the right of the circuit to delay the first operation on the qubit. Additionally, for quantum state tomography measurements, tomographic rotation is combined with an SQ gate at the end of the circuit, resulting in a U3 gate. To mitigate qubit-dephasing effects, these U3 gates are aligned to the left prior to measurement.

6. To suppress dephasing errors during idling, we incorporate dynamical decoupling (DD) gates. These DD gates are inserted within the circuit, which effectively suppresses the dephasing and thus enhances the overall performance.

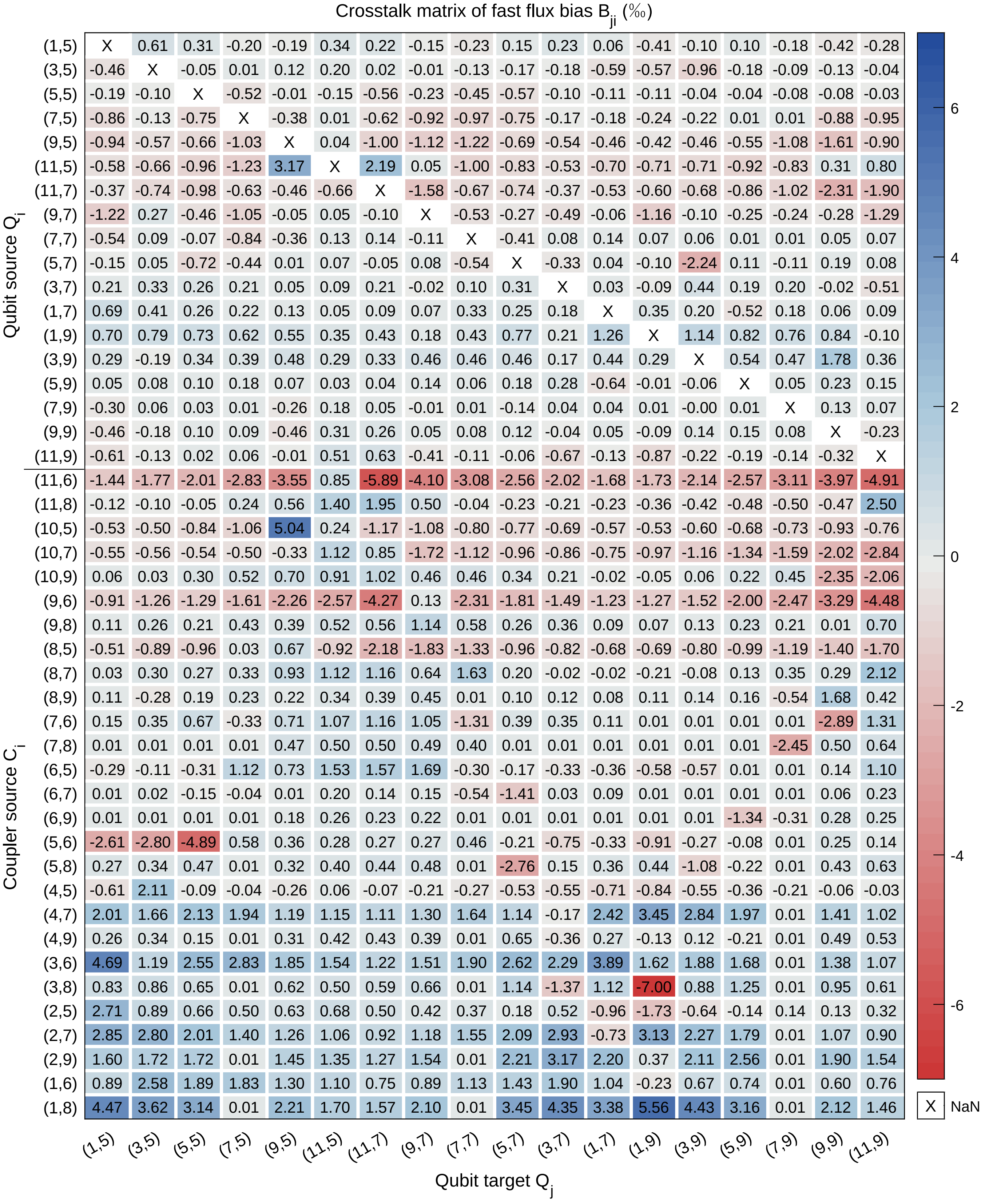

	(1,5)	(3,5)	(5,5)	(7,5)	(9,5)	(11,5)	(11,7)	(9,7)	(7,7)	(5,7)	(3,7)	(1,7)	(1,9)	(3,9)	(5,9)	(7,9)	(9,9)	(11,9)
(1,5)	X	0.61	0.31	-0.20	-0.19	0.34	0.22	-0.15	-0.23	0.15	0.23	0.06	-0.41	-0.10	0.10	-0.18	-0.42	-0.28
(3,5)	-0.46	X	-0.05	0.01	0.12	0.20	0.02	-0.01	-0.13	-0.17	-0.18	-0.59	-0.57	-0.96	-0.18	-0.09	-0.13	-0.04
(5,5)	-0.19	-0.10	X	-0.52	-0.01	-0.15	-0.56	-0.23	-0.45	-0.57	-0.10	-0.11	-0.11	-0.04	-0.04	-0.08	-0.08	-0.03
(7,5)	-0.86	-0.13	-0.75	X	-0.38	0.01	-0.62	-0.92	-0.97	-0.75	-0.17	-0.18	-0.24	-0.22	0.01	0.01	-0.88	-0.95
(9,5)	-0.94	-0.57	-0.66	-1.03	X	0.04	-1.00	-1.12	-1.22	-0.69	-0.54	-0.46	-0.42	-0.46	-0.55	-1.08	-1.61	-0.90
(11,5)	-0.58	-0.66	-0.96	-1.23	3.17	X	2.19	0.05	-1.00	-0.83	-0.53	-0.70	-0.71	-0.71	-0.92	-0.83	0.31	0.80
(11,7)	-0.37	-0.74	-0.98	-0.63	-0.46	-0.66	X	-1.58	-0.67	-0.74	-0.37	-0.53	-0.60	-0.68	-0.86	-1.02	-2.31	-1.90
(9,7)	-1.22	0.27	-0.46	-1.05	-0.05	0.05	-0.10	X	-0.53	-0.27	-0.49	-0.06	-1.16	-0.10	-0.25	-0.24	-0.28	-1.29
(7,7)	-0.54	0.09	-0.07	-0.84	-0.36	0.13	0.14	-0.11	X	-0.41	0.08	0.14	0.07	0.06	0.01	0.01	0.05	0.07
(5,7)	-0.15	0.05	-0.72	-0.44	0.01	0.07	-0.05	0.08	-0.54	X	-0.33	0.04	-0.10	-2.24	0.11	-0.11	0.19	0.08
(3,7)	0.21	0.33	0.26	0.21	0.05	0.09	0.21	-0.02	0.10	0.31	X	0.03	-0.09	0.44	0.19	0.20	-0.02	-0.51
(1,7)	0.69	0.41	0.26	0.22	0.13	0.05	0.09	0.07	0.33	0.25	0.18	X	0.35	0.20	-0.52	0.18	0.06	0.09
(1,9)	0.70	0.79	0.73	0.62	0.55	0.35	0.43	0.18	0.43	0.77	0.21	1.26	X	1.14	0.82	0.76	0.84	-0.10
(3,9)	0.29	-0.19	0.34	0.39	0.48	0.29	0.33	0.46	0.46	0.46	0.17	0.44	0.29	X	0.54	0.47	1.78	0.36
(5,9)	0.05	0.08	0.10	0.18	0.07	0.03	0.04	0.14	0.06	0.18	0.28	-0.64	-0.01	-0.06	X	0.05	0.23	0.15
(7,9)	-0.30	0.06	0.03	0.01	-0.26	0.18	0.05	-0.01	0.01	-0.14	0.04	0.04	0.01	-0.00	0.01	X	0.13	0.07
(9,9)	-0.46	-0.18	0.10	0.09	-0.46	0.31	0.26	0.05	0.08	0.12	-0.04	0.05	-0.09	0.14	0.15	0.08	X	-0.23
(11,9)	-0.61	-0.13	0.02	0.06	-0.01	0.51	0.63	-0.41	-0.11	-0.06	-0.67	-0.13	-0.87	-0.22	-0.19	-0.14	-0.32	X
(11,6)	-1.44	-1.77	-2.01	-2.83	-3.55	0.85	-5.89	-4.10	-3.08	-2.56	-2.02	-1.68	-1.73	-2.14	-2.57	-3.11	-3.97	-4.91
(11,8)	-0.12	-0.10	-0.05	0.24	0.56	1.40	1.95	0.50	-0.04	-0.23	-0.21	-0.23	-0.36	-0.42	-0.48	-0.50	-0.47	2.50
(10,5)	-0.53	-0.50	-0.84	-1.06	5.04	0.24	-1.17	-1.08	-0.80	-0.77	-0.69	-0.57	-0.53	-0.60	-0.68	-0.73	-0.93	-0.76
(10,7)	-0.55	-0.56	-0.54	-0.50	-0.33	1.12	0.85	-1.72	-1.12	-0.96	-0.86	-0.75	-0.97	-1.16	-1.34	-1.59	-2.02	-2.84
(10,9)	0.06	0.03	0.30	0.52	0.70	0.91	1.02	0.46	0.46	0.34	0.21	-0.02	-0.05	0.06	0.22	0.45	-2.35	-2.06
(9,6)	-0.91	-1.26	-1.29	-1.61	-2.26	-2.57	-4.27	0.13	-2.31	-1.81	-1.49	-1.23	-1.27	-1.52	-2.00	-2.47	-3.29	-4.48
(9,8)	0.11	0.26	0.21	0.43	0.39	0.52	0.56	1.14	0.58	0.26	0.36	0.09	0.07	0.13	0.23	0.21	0.01	0.70
(8,5)	-0.51	-0.89	-0.96	0.03	0.67	-0.92	-2.18	-1.83	-1.33	-0.96	-0.82	-0.68	-0.69	-0.80	-0.99	-1.19	-1.40	-1.70
(8,7)	0.03	0.30	0.27	0.33	0.93	1.12	1.16	0.64	1.63	0.20	-0.02	-0.02	-0.21	-0.08	0.13	0.35	0.29	2.12
(8,9)	0.11	-0.28	0.19	0.23	0.22	0.34	0.39	0.45	0.01	0.10	0.12	0.08	0.11	0.14	0.16	-0.54	1.68	0.42
(7,6)	0.15	0.35	0.67	-0.33	0.71	1.07	1.16	1.05	-1.31	0.39	0.35	0.11	0.01	0.01	0.01	0.01	-2.89	1.31
(7,8)	0.01	0.01	0.01	0.01	0.47	0.50	0.50	0.49	0.40	0.01	0.01	0.01	0.01	0.01	0.01	-2.45	0.50	0.64
(6,5)	-0.29	-0.11	-0.31	1.12	0.73	1.53	1.57	1.69	-0.30	-0.17	-0.33	-0.36	-0.58	-0.57	0.01	0.01	0.14	1.10
(6,7)	0.01	0.02	-0.15	-0.04	0.01	0.20	0.14	0.15	-0.54	-1.41	0.03	0.09	0.01	0.01	0.01	0.01	0.06	0.23
(6,9)	0.01	0.01	0.01	0.01	0.18	0.26	0.23	0.22	0.01	0.01	0.01	0.01	0.01	0.01	-1.34	-0.31	0.28	0.25
(5,6)	-2.61	-2.80	-4.89	0.58	0.36	0.28	0.27	0.27	0.46	-0.21	-0.75	-0.33	-0.91	-0.27	-0.08	0.01	0.25	0.14
(5,8)	0.27	0.34	0.47	0.01	0.32	0.40	0.44	0.48	0.01	-2.76	0.15	0.36	0.44	-1.08	-0.22	0.01	0.43	0.63
(4,5)	-0.61	2.11	-0.09	-0.04	-0.26	0.06	-0.07	-0.21	-0.27	-0.53	-0.55	-0.71	-0.84	-0.55	-0.36	-0.21	-0.06	-0.03
(4,7)	2.01	1.66	2.13	1.94	1.19	1.15	1.11	1.30	1.64	1.14	-0.17	2.42	3.45	2.84	1.97	0.01	1.41	1.02
(4,9)	0.26	0.34	0.15	0.01	0.31	0.42	0.43	0.39	0.01	0.65	-0.36	0.27	-0.13	0.12	-0.21	0.01	0.49	0.53
(3,6)	4.69	1.19	2.55	2.83	1.85	1.54	1.22	1.51	1.90	2.62	2.29	3.89	1.62	1.88	1.68	0.01	1.38	1.07
(3,8)	0.83	0.86	0.65	0.01	0.62	0.50	0.59	0.66	0.01	1.14	-1.37	1.12	-7.00	0.88	1.25	0.01	0.95	0.61
(2,5)	2.71	0.89	0.66	0.50	0.63	0.68	0.50	0.42	0.37	0.18	0.52	-0.96	-1.73	-0.64	-0.14	0.14	0.13	0.32
(2,7)	2.85	2.80	2.01	1.40	1.26	1.06	0.92	1.18	1.55	2.09	2.93	-0.73	3.13	2.27	1.79	0.01	1.07	0.90
(2,9)	1.60	1.72	1.72	0.01	1.45	1.35	1.27	1.54	0.01	2.21	3.17	2.20	0.37	2.11	2.56	0.01	1.90	1.54
(1,6)	0.89	2.58	1.89	1.83	1.30	1.10	0.75	0.89	1.13	1.43	1.90	1.04	-0.23	0.67	0.74	0.01	0.60	0.76
(1,8)	4.47	3.62	3.14	0.01	2.21	1.70	1.57	2.10	0.01	3.45	4.35	3.38	5.56	4.43	3.16	0.01	2.12	1.46

Supplementary Figure 12. **Crosstalk matrix of the fast flux bias.** Each qubit (coupler) is labeled by (x, y), which means it is located in the x-th column and y-th row in our device. Note that we neglect crosstalk to couplers in our experiments.

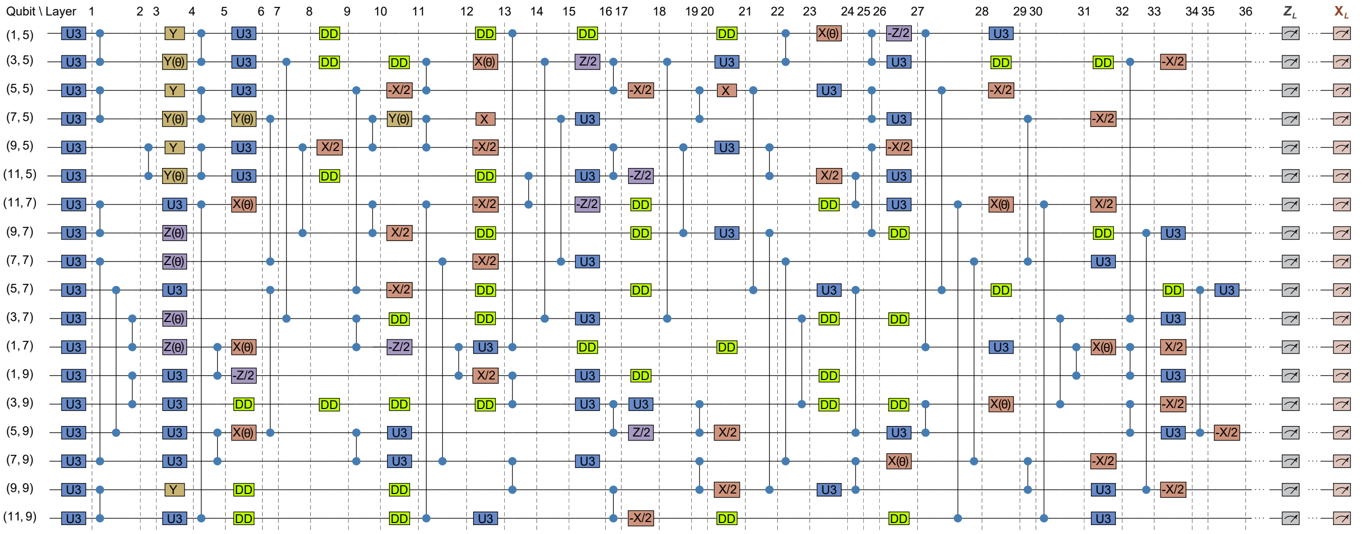

Supplementary Figure 13. **Experimental circuit to implement the Floquet unitary U_F at $B = 0.1$.** The first layer of the circuit encompasses the spin-flip driving designed for H_1, while the subsequent layers of the circuit simulate the evolution of the rotated surface code Hamiltonian H_2. U_F is composed of 15 SQ layers and 21 CZ layers. In total, it contains 117 single-qubit gates (excluding 40 dynamical decoupling (DD) gates) and 71 two-qubit CZ gates. We have three types of single-qubit gates: Clifford gates $\{X, Y, Z, \pm X/2, \pm Y/2, \pm Z/2\}$, parameterized rotation gates $\{X(\theta), Y(\theta), Z(\theta)\}$, and $\{U3(\alpha, \theta, \phi)\}$ gates. The DD gates are inserted in pairs. In our experiments, this circuit is repeated up to 20 cycles, for a total of about 2340 single-qubit gates and 1420 CZ gates. This equates to a time-domain sequence of $\sim$28.8 μs. Note that, at $B = 0$, single-qubit gates in the H_1 circuit are Clifford gates, and thus the whole circuit can be further simplified by merging some of them into the H_2 circuit.

F. Measurement of auto-correlation functions

While the definition of auto-correlation function in the main manuscript is not standard, it is the natural choice for comparing the auto-correlation function across different string operators. To unpack this idea, let us start by re-introducing the auto-correlation function:

$$A_L^{1/d}(t) = \text{sign}[\langle Z_L(0)Z_L(t)\rangle]|\langle Z_L(t)\rangle|^{1/d} \tag{18}$$

where Z_L is the string operator of length d. The motivation for this quantity is then two-fold: First, as emphasized in Supplementary Note 1.E, the string operator of a topologically ordered phase is expected to approach a steady state whose values decays exponentially with the length of the operator. As a result, if one directly compares $(-1)^t\langle Z_L(0)Z_L(t)\rangle$ for different lengths of strings, they will decay to very different values, even though the underlying physical phenomena is the same. Using a "geometric mean" weighting by taking d-th root of the value of the correlator normalizes this length decay and thus allows us to directly compare the dynamics of string operators of different sizes. Of course, this operation becomes ill-defined if the value is negative, so we separate the sign information from the amplitude information by multiplying, a posteriori, by the sign of the initial value. Second, one important feature of this quantity is that it is compatible with the single-spin auto-correlation function that is usually defined when studying other forms of time crystalline behavior [36]: $\Phi(t) = \langle\sigma(0)\sigma(t)\rangle$.

Having motivated how we have defined the auto-correlation function, we now turn to its measurement in the experiment. In our work, we focus on the nonlocal operator $M = Z_L$ which exhibits time-crystalline behavior. Since the initial state is a product state along z-basis, $|\langle Z_L(0)Z_L(t)\rangle| = |\langle Z_L(t)\rangle|$ and the auto-correlation function can be rewritten as $\text{sign}\left[\langle Z_L(0)Z_L(t)\rangle\right]|\langle Z_L(t)\rangle|^{1/d}$, as we describe in the main text. For the measured non-local X_L operators (see Fig. 2**a** in the main text), the values are conserved to nearly zero for initial states randomly chosen from the z-basis product states, and only the instantaneous values are plotted: $\text{sign}\left[\langle X_L(t)\rangle\right]|\langle X_L(t)\rangle|^{1/d}$.

We experimentally measured the dynamics of $Z_L(t)$ and $X_L(t)$ under the Floquet dynamics for up to 20 cycles. The expectation value of $Z_L(t)$, given by $\text{tr}\big(\rho(t)\prod_{k\in P_z}\sigma_k^z\big)$, is determined by the diagonal elements of the density matrix $\rho(t)$, which can be obtained by simultaneously measuring all relevant qubits in the z-basis. $X_L(t)$ is measured by applying a $-\pi/2$ rotation around the y-axis to each qubit (mapping the x-axis to the z-axis) before the final measurements along the z-axis. To ensure the statistical accuracy for the measurements of $Z_L(t)$ and $X_L(t)$, we repeat the state initialization, evolution sequence, and measurements 10,000 times, making the sample size large enough for accurately estimating probability distributions of 3 or 6 qubits. Their auto-correlation functions are then averaged over 24 realizations of parameters α_p, β_q, and initial product states.

Supplementary Note 3. Numerical simulations

For the experimentally-studied system size, we can numerically simulate the dynamics determined by specifically designed quantum circuits in our experiment. First, classical simulations allow us to evaluate the feasibility of the theoretical proposal by examining whether observables remain discernible after many Floquet cycles under realistic, noisy quantum gates. Second, we can deepen our understanding of our device performance by comparing the experimental results with simulation predictions that incorporate error models.

A. Error model and noisy simulation

We employ the Monte Carlo wavefunction method [37] to numerically simulate noisy circuits. The idea is to sample error operators according to a noise model and randomly insert them after each ideal gate. In this way, errors occur randomly in the circuit. We evolve the state vector along many quantum trajectories corresponding to many noise realizations. To obtain the value of the desired observable, we average over an ensemble of quantum trajectories, which resembles repeated measurements for evaluating the expectation value of an observable in real experiments. Notably, the Monte Carlo wavefunction method requires fewer computational resources compared to the master-equation approach, because it only stores state vectors of size 2^N during the calculation rather than density matrices of size $2^N \times 2^N$. In this context, we use the state-vector simulator provided by Qiskit for the numerical calculation of system dynamics. Qiskit provides several APIs to construct noise models that approximate the behavior of noisy circuits executed on real NISQ devices.

We model realistic errors with different quantum channels, and represent them with a probabilistic mixture of different operators. The corresponding parameters are estimated using the experimental benchmarks of gate errors and device performance. The following provides an introduction to the error model.

1. **Decoherence errors.** Due to interactions with the environment, energy relaxation and dephasing naturally occur in the dynamics can be described by the quantum channel

$$\mathcal{E}(\rho) = \begin{pmatrix} 1-\rho_{11}e^{-t/T_1} & \rho_{01}e^{-t/T_2} \\ \rho_{10}e^{-t/T_2} & \rho_{11}e^{-t/T_1} \end{pmatrix} = \sum_{i=0}^{3} M_i \rho M_i^\dagger, \tag{19}$$

where ρ is the density matrix of a single qubit with elements ρ_{ij} $(i,j=0,1)$. Here, T_1 represents the energy-relaxation time originating from energy exchange with the environment, and T_2 denotes the dephasing time characterizing the damping of off-diagonal terms of the density matrix. They satisfy the relation

$$\frac{1}{T_2} = \frac{1}{2T_1} + \frac{1}{T_\phi}, \tag{20}$$

where T_ϕ is the pure dephasing time, arising from non-dissipative interactions with the environment. This quantum channel can be written in terms of the Kraus operators

$$\begin{aligned} M_0 &= \sqrt{1-p_0-p_1}(|0\rangle\langle 0| + |1\rangle\langle 1|), \\ M_1 &= \sqrt{p_0}|0\rangle\langle 0|, \\ M_2 &= \sqrt{p_0}|0\rangle\langle 1|, \\ M_3 &= \sqrt{p_1}(|0\rangle\langle 0| - |1\rangle\langle 1|), \end{aligned} \tag{21}$$

which satisfy the normalization condition $\sum_{i=0}^{3} M_i^\dagger M_i = I$. The Kraus operator $M_0 \propto I$ indicates that the qubit remains intact with probability $1-p_0-p_1$. The pair of Kraus operators M_1 and M_2 describe spontaneous decay of the qubit from its excited state $|1\rangle$ to its ground state $|0\rangle$. This is realized in our simulations by randomly applying a reset operation to the qubit with probability $p_0 = 1-e^{-t/T_1}$. M_3 contributes an additional dephasing channel to the qubit. Together with the phase damping caused by M_1 and M_2, $M_{1,2,3}$ describe the total phase damping. M_3 is realized in our simulations by randomly applying σ^z operators with probability $p_1 = \frac{1}{2}e^{-t/T_1}[1-e^{-t(1/T_2-1/T_1)}]$. The two simulation parameters p_0 and p_1 are estimated using the average values of T_1 and T_2^{SE} reported in Supplementary Note 2.A, while the value of t is set by the averaged time required to apply an SQ (CZ) layer.

2. **Depolarizing errors.** The depolarizing channel is defined as

$$\mathcal{E}(\rho) = (1-e_p)\,\rho + \frac{e_p}{4^d-1}\sum_{\mu\neq 0} P_\mu \rho P_\mu, \tag{22}$$

where d is the number of qubits, ρ is a d-qubit density operator, $P_\mu \in \{I, X, Y, Z\}^{\otimes d}$ is the tensor product of Pauli gates, and e_p denotes the Pauli error per cycle. We use the depolarizing channel to account for errors caused by imperfect control of the system, such as gate control errors and crosstalk errors. It is realized in our simulations by applying a randomly chosen non-identity Pauli string with probability $e_p/(4^d - 1)$.

Using the error model above, we perform numerical simulations to verify the observed results in the main text. The decoherence error for each SQ (CZ) layer, quantified by p_0 and p_1 in the model, is estimated with T_1 and T_2 fixed by the average measured T_1, T_2^{SE} values (see Supplementary Figure 9), and with t set by the pulse duration corresponding to an SQ or CZ layer, depending on whether the error channel is being applied after a single- or two-qubit gate layer. The average pulse duration is about 24.0 ns for an SQ layer and 62.6 ns (52.5 ns) for a CZ layer in circuits with (without) eigenstate preparation. Then, the depolarization error rate e_p for each type of gate is estimated by subtracting the decoherence error rate from the median Pauli error rate ϵ_p estimated in Supplementary Note 2.B. Here, Pauli errors are characterized using XEB experiments (see Supplementary Figure 10), whose median value is $\epsilon_p \sim 0.48 \times 10^{-3}$ for a single-qubit gate, 0.64×10^{-2} for qubits involved in a CZ gate, and 1.37×10^{-3} (1.10×10^{-3}) for qubits that are idle during a CZ layer, in experimental circuits with (without) eigenstate preparation. Simulation results using the error sources above are shown in Fig. 2a and Fig. 4b, c, and e of the main text and exhibit good agreement with the experiments.

B. Numerical comparison with echo sequence

One commonly used technique to distinguish the decay of auto-correlation functions caused by external decoherence and that resulting from internal thermalization is the "echo" circuit $U_{\mathrm{echo}} = (U_F^\dagger)^t (U_F)^t$ [17, 38], which reverses the Floquet time evolution after time step t. However, its implementation requires a doubling of the length of the experimental sequence, resulting in 1440 layers of quantum gates and a total sequence time of 57.6 μs. This requirement is beyond the reach of our current NISQ device.

Another general method to evaluate the effects of external decoherence is to measure gate errors and decoherence times with standard benchmarks, such as cross-entropy benchmarking (XEB), energy relaxation time T_1, and spin-echo dephasing

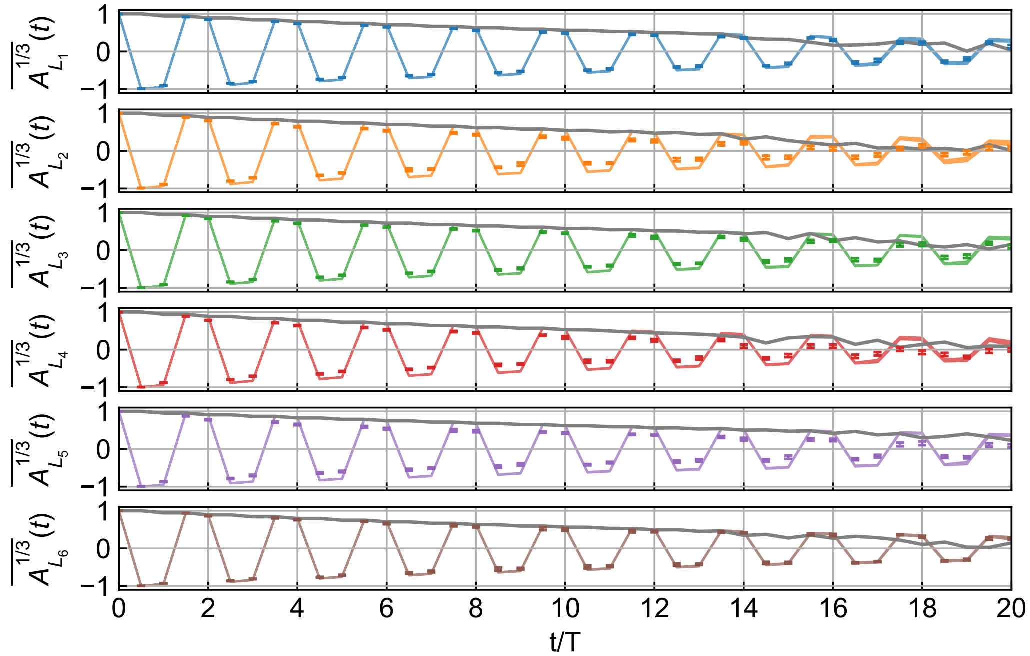

Supplementary Figure 14. Numerical results of the echoed dynamics for $\{Z_{L_i}\}$ under $U_{\mathrm{echo}} = (U_F^\dagger)^t (U_F)^t$. The auto-correlation functions $A^{1/3(\mathrm{echo})}$ for U_{echo} (grey lines) accurately captures the observed decay of $A^{1/3}$ in both our previous numerical simulation (colored lines) as well as experimentally measured data (dots). Values of $A^{1/3(\mathrm{echo})}$ are averaged over the same 24 random realizations as that under U_F. The plotted auto-correlation functions $A^{1/3}$ for U_F replicate the data points in Fig. 2a of the main text.

time T_2^{SE}, and then perform noisy numerical simulations with these error sources. In our work, we carefully benchmarked these errors (Supplementary Note 2.B) and used open source framework (IBM Qiskit) to perform the numerical simulations (Supplementary Note 3.A). The numerical simulation of the experiment is in excellent agreement with the experimental data (Fig. 2 of the main text), indicating a thorough understanding of the decoherence mechanisms in our experiments.

We complement this analysis by numerically simulating the echoed sequence $[U_{\mathrm{echo}} = (U_F^\dagger)^t(U_F)^t]$. Supplementary Figure 14 displays the numerical results of the $\{Z_{L_i}\}$ operators dynamics (grey lines), the auto-correlation functions of which are defined as $A_L^{1/3(\mathrm{echo})}(t) = \mathrm{sign}[\langle Z_L(0)Z_L(t)\rangle]|\langle Z_L(t)\rangle|^{1/(3\times 2)}$ (note that an additional square root is taken to account for the twice longer sequence of U_{echo}). We find that $A^{1/3(\mathrm{echo})}$ accurately captures the observed decay in both our previous numerical simulation as well as experimentally measured data, providing a strong indication that the observed decay is dominated by external sources of decoherence rather than internal thermalization. These results further emphasize the robustness of the topologically ordered time crystalline behavior observed.

---

[1] X.-G. Wen, Colloquium: Zoo of quantum-topological phases of matter, Rev. Mod. Phys. **89**, 041004 (2017).
[2] T. B. Wahl, B. Han, and B. Béri, Topologically ordered time crystals, arXiv:2105.09694 (2021).
[3] X.-L. Qi and S.-C. Zhang, Topological insulators and superconductors, Rev. Mod. Phys. **83**, 1057 (2011).
[4] T. Senthil, Symmetry-Protected Topological Phases of Quantum Matter, Annu. Rev. Condens. Matter Phys. **6**, 299 (2015).
[5] K. Fujii, Topological stabilizer codes, in Quantum Computation with Topological Codes: From Qubit to Topological Fault-Tolerance (Springer Singapore, 2015) pp. 56–85.
[6] A. G. Fowler, M. Mariantoni, J. M. Martinis, and A. N. Cleland, Surface codes: Towards practical large-scale quantum computation, Phys. Rev. A **86**, 032324 (2012).
[7] S. Krinner, N. Lacroix, A. Remm, A. Di Paolo, E. Genois, C. Leroux, C. Hellings, S. Lazar, F. Swiadek, J. Herrmann, et al., Realizing repeated quantum error correction in a distance-three surface code, Nature **605**, 669 (2022).
[8] R. Acharya, I. Aleiner, R. Allen, T. I. Andersen, M. Ansmann, F. Arute, K. Arya, A. Asfaw, J. Atalaya, R. Babbush, et al., Suppressing quantum errors by scaling a surface code logical qubit, Nature **614**, 676 (2023).
[9] D. Bluvstein, S. J. Evered, A. A. Geim, S. H. Li, H. Zhou, T. Manovitz, S. Ebadi, M. Cain, M. Kalinowski, D. Hangleiter, et al., Logical quantum processor based on reconfigurable atom arrays, Nature , 1 (2023).
[10] C. Horsman, A. G. Fowler, S. Devitt, and R. V. Meter, Surface code quantum computing by lattice surgery, New J. Phys. **14**, 123011 (2012).
[11] A. Kitaev and J. Preskill, Topological Entanglement Entropy, Phys. Rev. Lett. **96**, 110404 (2006).
[12] E. V. H. Doggen, I. V. Gornyi, A. D. Mirlin, and D. G. Polyakov, Slow many-body delocalization beyond one dimension, Phys. Rev. Lett. **125**, 155701 (2020).
[13] I.-D. Potirniche, S. Banerjee, and E. Altman, Exploration of the stability of many-body localization in $d > 1$, Phys. Rev. B **99**, 205149 (2019).
[14] W. De Roeck and J. Z. Imbrie, Many-body localization: Stability and instability, Philos. Trans. R. Soc. Math. Phys. Eng. Sci. **375**, 20160422 (2017).
[15] K. G. Wilson, Confinement of quarks, Phys. Rev. D **10**, 2445 (1974).
[16] B. Bauer and C. Nayak, Area laws in a many-body localized state and its implications for topological order, J. Stat. Mech.: Theory Exp. **2013** (09), P09005.
[17] X. Zhang, W. Jiang, J. Deng, K. Wang, J. Chen, P. Zhang, W. Ren, H. Dong, S. Xu, Y. Gao, F. Jin, X. Zhu, Q. Guo, H. Li, C. Song, A. V. Gorshkov, T. Iadecola, F. Liu, Z.-X. Gong, Z. Wang, et al., Digital quantum simulation of Floquet symmetry-protected topological phases, Nature **607**, 468 (2022).
[18] M. Levin and X.-G. Wen, Detecting topological order in a ground state wave function, Phys. Rev. Lett. **96**, 110405 (2006).
[19] M. Cerezo, A. Arrasmith, R. Babbush, S. C. Benjamin, S. Endo, K. Fujii, J. R. McClean, K. Mitarai, X. Yuan, L. Cincio, and P. J. Coles, Variational quantum algorithms, Nat. Rev. Phys. **3**, 625 (2021).
[20] W. Li, Z. Lu, and D.-L. Deng, Quantum Neural Network Classifiers: A Tutorial, SciPost Phys. Lect. Notes , 61 (2022).
[21] Z. Lu, P.-X. Shen, and D.-L. Deng, Markovian Quantum Neuroevolution for Machine Learning, Phys. Rev. Appl. **16**, 044039 (2021).
[22] K. J. Satzinger, Y.-J. Liu, A. Smith, C. Knapp, M. Newman, C. Jones, Z. Chen, C. Quintana, X. Mi, A. Dunsworth, et al., Realizing topologically ordered states on a quantum processor, Science **374**, 1237 (2021).
[23] F. Yan, P. Krantz, Y. Sung, M. Kjaergaard, D. L. Campbell, T. P. Orlando, S. Gustavsson, and W. D. Oliver, Tunable coupling scheme for implementing high-fidelity two-qubit gates, Phys. Rev. Appl. **10**, 054062 (2018).
[24] P. V. Klimov, J. Kelly, Z. Chen, M. Neeley, A. Megrant, B. Burkett, R. Barends, K. Arya, B. Chiaro, Y. Chen, et al., Fluctuations of energy-relaxation times in superconducting qubits, Phys. Rev. Lett. **121**, 090502 (2018).
[25] C. Neill, P. Roushan, K. Kechedzhi, S. Boixo, S. V. Isakov, V. Smelyanskiy, A. Megrant, B. Chiaro, A. Dunsworth, K. Arya, R. Barends, et al., A blueprint for demonstrating quantum supremacy with superconducting qubits, Science **360**, 195 (2018).
[26] R. Barends, J. Kelly, A. Megrant, A. Veitia, D. Sank, E. Jeffrey, T. C. White, J. Mutus, A. G. Fowler, B. Campbell, et al., Superconducting quantum circuits at the surface code threshold for fault tolerance, Nature **508**, 500 (2014).
[27] S. Boixo, S. V. Isakov, V. N. Smelyanskiy, R. Babbush, N. Ding, Z. Jiang, M. J. Bremner, J. M. Martinis, and H. Neven, Characterizing quantum supremacy in near-term devices, Nat. Phys. **14**, 595 (2018).
[28] F. Arute, K. Arya, R. Babbush, D. Bacon, J. C. Bardin, R. Barends, R. Biswas, S. Boixo, F. G. S. L. Brandao, D. A. Buell, et al., Quantum

supremacy using a programmable superconducting processor, Nature **574**, 505 (2019).
[29] P. D. Nation, H. Kang, N. Sundaresan, and J. M. Gambetta, Scalable mitigation of measurement errors on quantum computers, PRX Quantum **2**, 040326 (2021).
[30] Y. Sung, L. Ding, J. Braumüller, A. Vepsäläinen, B. Kannan, M. Kjaergaard, A. Greene, G. O. Samach, C. McNally, D. Kim, et al., Realization of high-fidelity cz and zz-free iswap gates with a tunable coupler, Phys. Rev. X **11**, 021058 (2021).
[31] A. Kissinger and J. van de Wetering, PyZX: Large scale automated diagrammatic reasoning, Electronic Proceedings in Theoretical Computer Science **318**, 229 (2020).
[32] A. Kissinger and J. van de Wetering, Reducing the number of non-clifford gates in quantum circuits, Phys. Rev. A **102**, 022406 (2020).
[33] Qiskit contributors, Qiskit: An open-source framework for quantum computing (2023).
[34] Cirq Developers, Cirq (2022), See full list of authors on Github: https://github .com/quantumlib/Cirq/graphs/contributors.
[35] D. C. McKay, C. J. Wood, S. Sheldon, J. M. Chow, and J. M. Gambetta, Efficient z gates for quantum computing, Phys. Rev. A **96**, 022330 (2017).
[36] D. V. Else, C. Monroe, C. Nayak, and N. Y. Yao, Discrete Time Crystals, Annu. Rev. Condens. Matter Phys. **11**, 467 (2020).
[37] K. Mølmer, K. Berg-Sørensen, Y. Castin, and J. Dalibard, A monte carlo wave function method in quantum optics, in Optical Society of America Annual Meeting (Optica Publishing Group, 1992) p. MFF1.
[38] X. Mi, M. Ippoliti, C. Quintana, A. Greene, Z. Chen, J. Gross, F. Arute, K. Arya, J. Atalaya, R. Babbush, J. C. Bardin, J. Basso, A. Bengtsson, A. Bilmes, A. Bourassa, L. Brill, M. Broughton, B. B. Buckley, D. A. Buell, B. Burkett, et al., Time-crystalline eigenstate order on a quantum processor, Nature **601**, 531 (2022).
